# A blueprint of mammalian cortical connectomes

**DOI:** 10.1371/journal.pbio.2005346

**Published:** 2019-03-22

**Authors:** Alexandros Goulas, Piotr Majka, Marcello G. P. Rosa, Claus C. Hilgetag

**Affiliations:** 1 Institute of Computational Neuroscience, University Medical Center Hamburg-Eppendorf, Hamburg University, Hamburg, Germany; 2 Laboratory of Neuroinformatics, Nencki Institute of Experimental Biology of Polish Academy of Sciences, Warsaw, Poland; 3 ARC Centre of Excellence for Integrative Brain Function, Monash University Node, Monash University, Clayton, Australia; 4 Department of Physiology, Biomedicine Discovery Institute, Monash University, Clayton, Australia; 5 Department of Health Sciences, Boston University, Boston, Massachusetts, United States of America; University of Oxford, United Kingdom of Great Britain and Northern Ireland

## Abstract

The cerebral cortex of mammals exhibits intricate interareal wiring. Moreover, mammalian cortices differ vastly in size, cytological composition, and phylogenetic distance. Given such complexity and pronounced species differences, it is a considerable challenge to decipher organizational principles of mammalian connectomes. Here, we demonstrate species-specific and species-general unifying principles linking the physical, cytological, and connectional dimensions of architecture in the mouse, cat, marmoset, and macaque monkey. The existence of connections is related to the cytology of cortical areas, in addition to the role of physical distance, but this relation is attenuated in mice and marmoset monkeys. The cytoarchitectonic cortical gradients, and not the rostrocaudal axis of the cortex, are closely linked to the laminar origin of connections, a principle that allows the extrapolation of this connectional feature to humans. Lastly, a network core, with a central role under different modes of network communication, characterizes all cortical connectomes. We observe a displacement of the network core in mammals, with a shift of the core of cats and macaque monkeys toward the less neuronally dense areas of the cerebral cortex. This displacement has functional ramifications but also entails a potential increased degree of vulnerability to pathology. In sum, our results sketch out a blueprint of mammalian connectomes consisting of species-specific and species-general links between the connectional, physical, and cytological dimensions of the cerebral cortex, possibly reflecting variations and persistence of evolutionarily conserved mechanisms and cellular phenomena. Our framework elucidates organizational principles that encompass but also extend beyond the wiring economy principle imposed by the physical embedding of the cerebral cortex.

## Author summary

The cerebral cortex is wired in a highly intricate manner and exhibits striking differences across mammals—for instance, in overall size and number of neurons. Here, we uncover common, but also species-specific, principles that link the physical, cellular, and connectional architecture of mouse, cat, and monkey brains. Commonalities allow the extrapolation of features to further, unexamined species, such as humans, whereas the species-specific principles point at potential functional differences, but also varied degrees of vulnerability, of mammalian brains. The observed unifying principles may reflect variations of evolutionarily conserved neurodevelopmental mechanisms. In sum, we sketch out a blueprint of mammalian cortical organization that elucidates the links between the physical, cytological, and connectional architecture. Our results indicate that caution is warranted when translating findings from one mammalian species to another, since some, but not all, cortical organizational properties are common across the mammalian spectrum.

## Introduction

**Fig 1:**
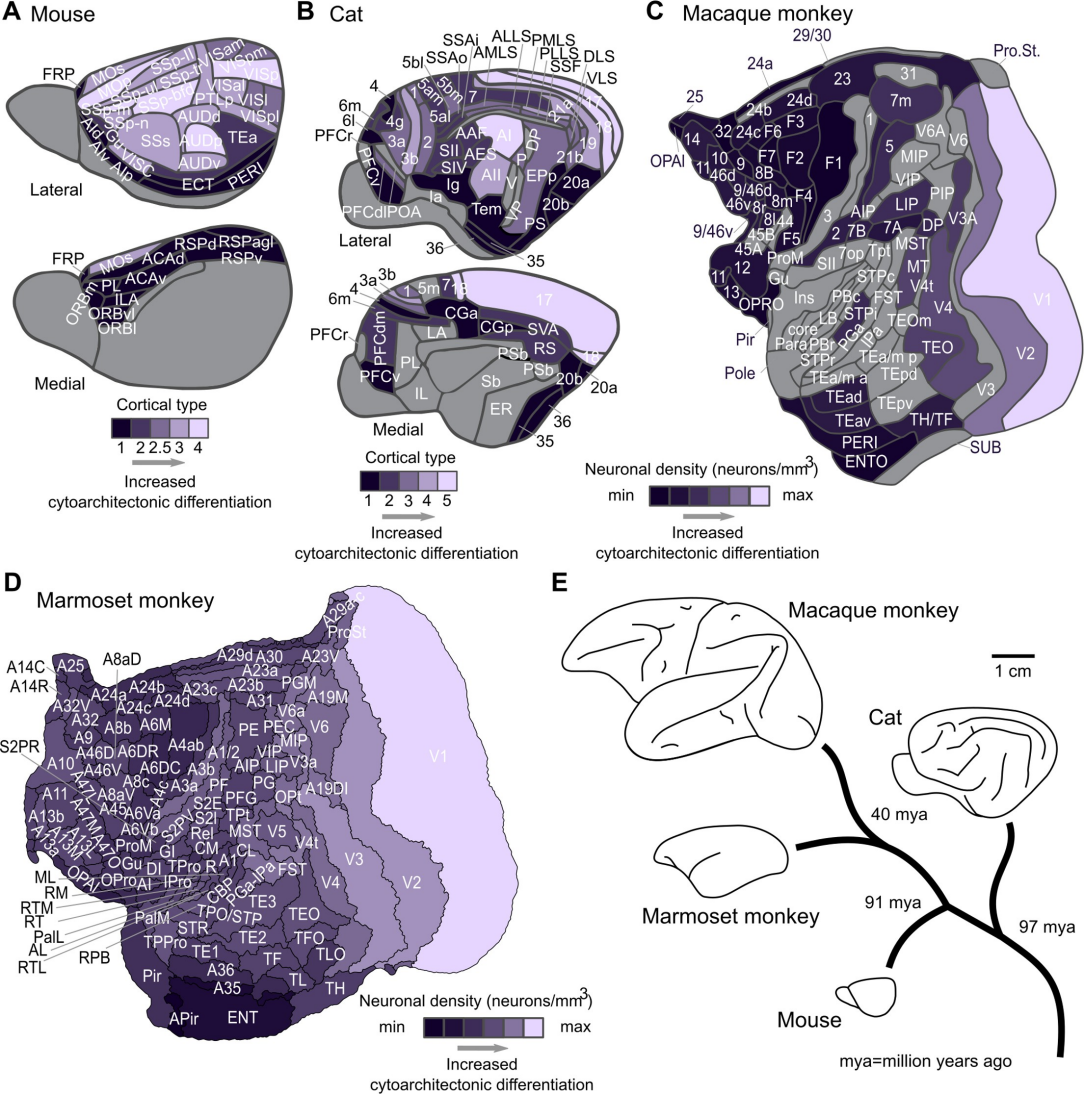
Mammalian cerebral cortices. (A) Mouse, (B) cat, (C) macaque monkey, and (D) marmoset monkey cortex. Cortical areas are shown with their respective cytoarchitectonic status dictated by cortical types (mouse and cat) or neuronal density per mm^3^ (marmoset and macaque monkey). Cortical types define an ordinal scale from cytoarchitectonically less differentiated areas, which correspond to overall less neuronally dense areas (lower cortical types), to cytoarchitectonically more differentiated areas, which correspond to overall more neuronally dense areas (higher cortical types). Note that there is no one-to-one correspondence of the cortical types for the mouse and cat cortex. Each scale denotes degrees of cytoarchitectonic differentiation within each species. (E) Illustration of cortex size differences and phylogenetic relations of the examined species. See S1 Table for full names of the cortical areas. max, maximum; min, minimum.

Mapping and understanding the wiring of the cerebral cortex at the micro-, meso-, and macroscale level is a central challenge in neuroscience [1–9]. Extensive studies have mapped the structural connections among cortical areas—that is, the macroscale connectional architecture—in different mammals, such as cats [3], mice [6, 7], and macaque and marmoset monkeys [8, 10]. These studies have uncovered a characteristic pattern of cortico-cortical connections among cortical areas, providing the structural scaffold for the communication of cortical areas, which is essential for cognition and behavior [11–13]. Moreover, invasive tract-tracing studies in mammals have uncovered a graded variation in the laminar origin of connections [14–16], a connectional feature related to physiological properties of long-range connections [12], central to contemporary theories of brain structure and function [17, 18] and the basis of the so-called hierarchical arrangement of the areas of the cerebral cortex [19]. From a network topology standpoint—that is, the arrangement of connections between the distinct areas of the cortex—cortical connectomes possess a tightly interconnected structural core [20, 21], a network topology that is considered important for flexible behavior and large-scale functional integration [22]. Moreover, mammalian species exhibit a divergent evolutionary history of millions of years, as well as pronounced differences with respect to, for instance, brain size, number of neurons, and duration of neurogenesis [23–28] (Fig 1). Given the intricate wiring configuration of the cortex and such pronounced differences, is it possible to decipher unifying principles that link the connectional architecture with other dimensions of cortical architecture and thus sketch out a blueprint of the cortical organization of mammals?

A principle related to the existence of connections is the wiring cost principle. Specifically, nearby areas are more likely to be connected than remote areas [21, 29–31]. However, wiring cost, reflected in the physical distance between cortical areas, does not fully explain the existence of connections [21, 32, 33]. Qualitative observations in the macaque monkey cortex suggest that the existence of connections is closely related to the gradients of cytoarchitectonic differentiation of the cerebral cortex, specifically to the similarity of the degree of cytoarchitectonic differentiation of cortical areas [34]. Gradients of cytoarchitectonic differentiation are formed by spatially ordered changes in the cytological composition of areas, including the appearance of the granular layer (layer IV) and the increase of its neuronal density and width, as well as the successive distinguishability and increase of the neuronal density of upper (supragranular) layers compared to lower (infragranular) layers [16, 27, 35–38]. Systematic studies in different mammalian species have demonstrated that the similarity of cytoarchitectonic differentiation of cortical areas, above and beyond their physical distance, is closely related to the existence of connections, suggesting a common wiring principle of mammalian cortices [30, 31, 33, 39]. It is, however, unknown if this wiring principle is manifested in a species-specific manner and how general it is across the mammalian phylogeny.

With respect to the systematic shifts of the laminar origin of connections, two main explanations have been put forward. On the one hand, a framework postulates that the graded shift of the laminar origin of connections, from predominantly infragranular to predominantly supragranular, giving rise to “feedback” and “feedforward” type of connections, respectively, is manifested across the rostrocaudal axis of the brain [14, 40–42]. On the other hand, a cytoarchitecture-based framework has emphasized the central role of cortical cytoarchitectonic gradients in relation to the gradual shifts of the laminar origin of connections [15, 30, 33, 39, 43]. Therefore, a conjoint examination of these alternative frameworks in different mammalian cortices is needed for deciphering the central dimension of cortical organization that is related to the graded shifts of the laminar origin of connections.

From a network topology standpoint—that is, the arrangement of connections between the different areas of the cortex—a core–periphery structure characterizes the mouse and macaque monkey cortex [20, 21]. The core–periphery structure corresponds to two sets of areas, a tightly interconnected set of areas constituting the core and the rest of the areas constituting the periphery. This network configuration is central to theories of animal cognition [22]. In the macaque monkey, the core–periphery division is also reflected in the cytoarchitecture of the cortex, thus offering a unifying principle linking network topology and cytology by elucidating the cellular composition of topologically central cortical areas and the potential neurodevelopmental mechanisms leading to their central role in the cortical connectome [33]. Therefore, it is important to elucidate the species-general or species-specific nature of the relation of the core–periphery structure to the cytology of the cortex across different mammals.

Here, we examine the connectomes of the mouse, cat, and macaque and marmoset monkey. We relate the connectional, cytoarchitectonic, and physical dimensions of the cerebral cortex, thus highlighting unifying principles that link the different dimensions of cortical architecture. These principles are manifested in a species-general but also species-specific manner, distinguishing the mouse and the marmoset monkey from the cat and the macaque monkey cortex. Commonalities allow the extrapolation of connectional features to unexamined species, such as humans, whereas the species-specific principles point at potential functional differences across species and indicate varied degrees of vulnerability to pathology. The observed unifying principles may reflect variations of evolutionary conserved neurodevelopmental mechanisms.

## Results

### Cytoarchitectonic similarity relates to the existence of connections in a species-specific manner

Extensive cytoarchitectonic and connectome data as well as information on the physical distance between cortical areas of mouse, cat, and marmoset and macaque monkey cortices were used in the analyses [8, 10, 30, 31, 33, 44, 45]. For the mouse and cat cortex, the cytoarchitectonic differentiation of areas was assessed qualitatively by defining an ordinal scale of cortical types based on Nissl-stained sections [30, 31]. Cortical types reflect a multidimensional characterization of the cytoarchitectonic differentiation of cortical areas, based primarily on the density of neurons in the different cortical layers, as well as the appearance, neuronal density, and thickness of layer IV [39]. Low cortical types—that is, less differentiated and overall less neuronally dense areas—are not clearly laminated, and layer IV is absent or only weakly present. By contrast, high cortical types—that is, more differentiated and overall more neuronally dense areas—are clearly laminated, with a clearly defined layer IV. By these criteria, the highest cortical type corresponds to areas such as the primary visual cortex [30, 39]. Therefore, cortical areas constitute a cortical spectrum of cytoarchitectonic differentiation, ranging from less to more differentiated and, thus, overall neuronally dense cortical areas (Fig 1). The cytoarchitectonic differentiation of the marmoset and macaque monkey cortical areas was assessed quantitatively by their overall neuronal density—that is, the number of neurons per mm^3^. Neuronal density constitutes a fingerprint of the cytoarchitectonic status of cortical areas [33, 36, 37]. For the macaque monkey, Nissl- and NeuN-stained material was used [33]. For the marmoset monkey, NeuN-stained material was used [44]. Qualitative assessment of cytoarchitectonic differentiation of the areas of the macaque monkey, based on cortical types, was used as a control analysis. We used the most-comprehensive available cortical connectomes of the mouse [7, 21], cat [3], and marmoset [10] and macaque monkey [8]. We used the geodesic or Euclidean distance between the barycenters of the cortical areas as a measure of their physical distance [8, 10, 21, 31]. In the absence of a stereotaxic atlas with the parcellation scheme of Scannell and colleagues [3] for the cat cortex, physical distance between areas of the cat cortex was defined as the number of areas separating a pair of areas [30].

**Fig 2:**
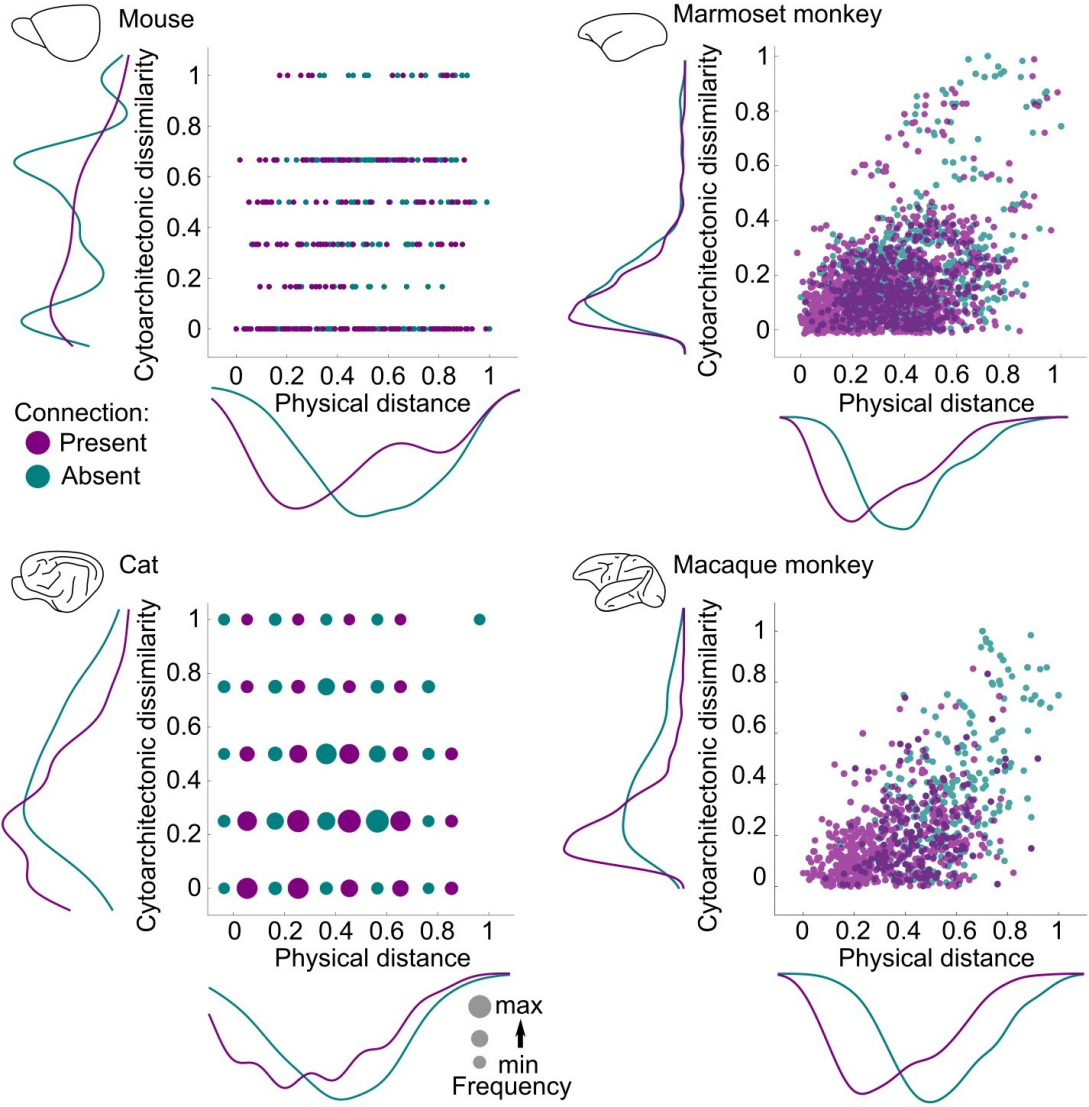
Existence of connections in relation to physical distance and cytoarchitectonic similarity of cortical areas. The existence of connections is reflected in the cytoarchitectonic similarity of cortical areas and the physical distance between them, as is evident in the density plots of each axis. Present connections span short distances and link cytoarchitectonically similar areas, whereas the opposite holds for absent connections. However, for the marmoset monkey, conjoint multivariate examination of cytoarchitectonic similarity and physical distance shows a lack of statistical significance between cytoarchitectonic similarity and existence of connections (S2 Fig), thus pointing out a species-species manifestation of the relation between cytoarchitecture and existence of connections. Depicted cytoarchitectonic similarity and physical distance values are the result of a linear rescaling to the 0–1 interval. Note that for the cat cortex, both physical distance and cytoarchitectonic similarity are ordinal scales, and thus the frequency of presence or absence of connections for each pair of the ordinal values is depicted. Note as well that for the cat cortex, data points correspond only to connections with known status (present or absent) [30]; hence, no data points for certain physical distance and cytoarchitectonic similarity combinations are depicted. max, maximum; min, minimum.

The presence or absence of a connection was viewed against two dimensions of cortical organization—that is, the cytoarchitectonic and physical dimension (Fig 2). We verified that connections that are present span shorter distances than absent connections (statistical energy test: 0.17, 0.74, 0.03, 0.33 for the mouse, cat, and marmoset and macaque monkey, respectively; all *p* < 0.001). Moreover, connections that are present involve pairs of areas with more-similar cytoarchitecture than areas that are not connected (statistical energy test: 0.32, 0.29, 0.16, 0.23 for the mouse, cat, and marmoset and macaque monkey, respectively; all *p* < 0.001). Similarity of cytoarchitecture was assessed as the absolute difference of the cortical type or neuronal density of a pair of areas. Fig 2 summarizes these findings, demonstrating that cytoarchitectonic similarity of cortical areas and their physical distance relates to the existence of connections. Using an alternative dataset for mouse connectivity [21] and qualitative assessment of the cytoarchitectonic status of the areas for the macaque monkey cortex led to similar qualitative results (S1 Fig).

Conjoint examination of the role of cytoarchitectonic similarity and physical distance to existence of connections with multivariate logistic regression revealed a statistically significant contribution of both factors in all species with the exception of the marmoset monkey, in which cytoarchitectonic similarity did not reach statistical significance (S2 Fig). Thus, in the marmoset monkey, cytoarchitectonic similarity does not relate, above and beyond physical distance, to the pattern of existence of connections among cortical areas. This discrepancy constitutes the first species-specific manifestation of the relation of cytoarchitecture and connectivity. Control analyses for the species for which an ordinal scale was used in assessing the cytoarchitectonic status of cortical areas—that is, the cat and mouse—revealed that the relation of cytoarchitectonic similarity and existence of connections was robust to the exact assignments of cortical types to areas, as well as the exact range of the ordinal scale used for the qualitative evaluation of cytoarchitectonic differentiation in these species (S3 Fig).

To further investigate species-specific relations of cytoarchitecture and connectivity, we performed a logistic regression analysis for the mammals that showed a significant relation between cytoarchitecture and connectivity in the multivariate logistic regression analysis—that is, the mouse, cat, and macaque monkey. For each pair of these mammals, a model was estimated with existence of connections as the binary dependent variable and cytoarchitectonic similarity and distance as regressors. For investigating species-specific effects, a further regressor coding for the different species and their interaction with cytoarchitectonic similarity was added. Coefficients from the logistic regression denote the impact of each regressor on the probability of finding a connection between a pair of areas, as well as the dependence of such an effect on the interaction of the regressors. It should be noted that our approach does not require the establishment of area homologies across species, since the cross-species analysis relies on pairs of cortical areas and examines the factors related to the presence or absence of a connection between each pair of areas, irrespective of potential homologies or absence thereof.

These analyses showed that the effect of cytoarchitectonic similarity on the existence of connections was significant in all cases, but its role was different when the mouse was compared with the cat and macaque monkey. For the mouse versus macaque monkey analysis, the coefficients were distance = −2.74, cytoarchitectonic similarity = −0.80, and species by cytoarchitectonic similarity = −2.47 (all *p* < 0.0001). The inclusion of the species by cytoarchitectonic similarity interaction significantly improved the model fit, as indicated by a likelihood ratio (LR) test (LR = 16.95, *p* < 0.001). For the mouse versus cat analysis, the coefficients were distance = −1.67, cytoarchitectonic similarity = −0.90, and species by cytoarchitectonic similarity = −1.77 (all *p* < 0.0001). The inclusion of the species by cytoarchitectonic similarity interaction significantly improved the model fit, as indicated by an LR test (LR = 20.55, *p* < 0.001). For the cat versus macaque monkey analysis, the coefficients were distance = −2.70 and cytoarchitectonic similarity = −2.76, (both *p* < 0.001), but the interaction between the species and cytoarchitectonic similarity regressors was not significant (species by cytoarchitectonic similarity = −0.53, *p* > 0.1).

**Fig 3:**
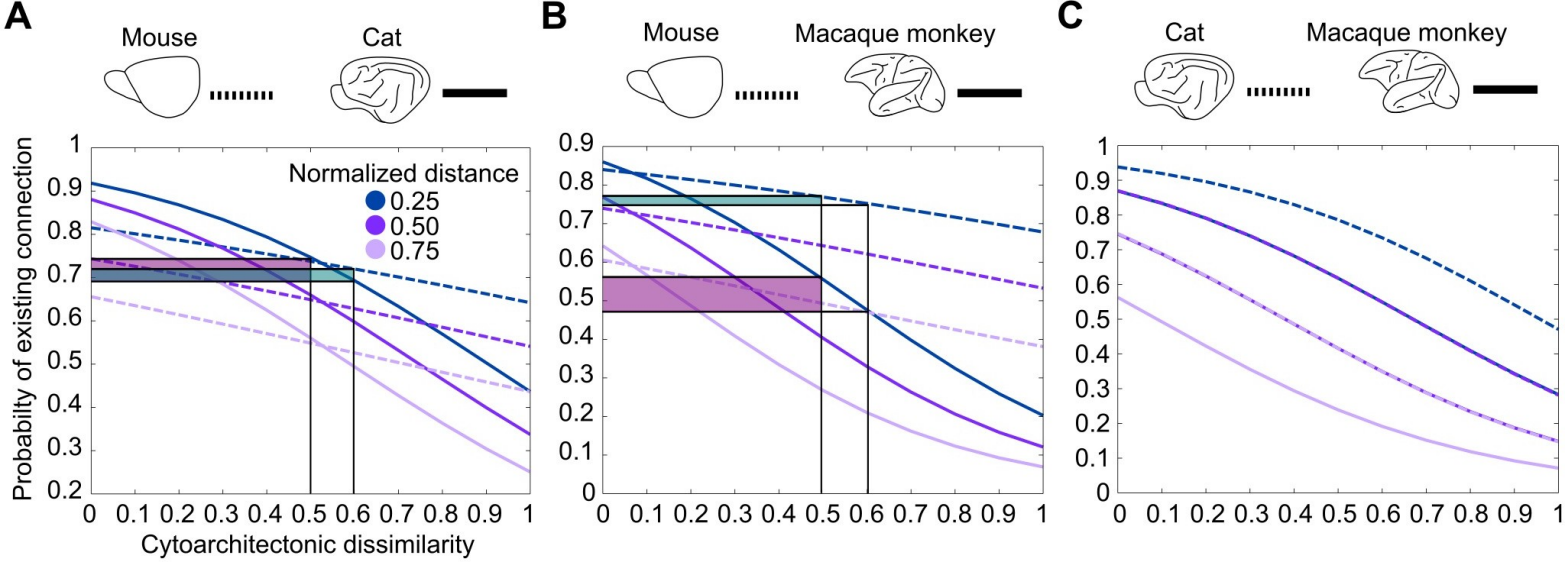
Cytoarchitectonic similarity relates to the existence of connections in a species-specific manner. (A) Increasing cytoarchitectonic dissimilarity of cortical areas entails a decrease in the probability of the existence of a connection. This decrease is more pronounced for the cat when compared to the mouse, as indicated by the larger probability decrease (shaded areas) for the same increase of cytoarchitectonic dissimilarity. (B) Same relation as in (A), but for the comparison of mouse versus macaque monkey. The decrease of the probability of the existence of a connection is more pronounced for the macaque monkey when compared to the mouse. (C) Same relation as in (A), but for the comparison of cat versus macaque monkey. In this comparison, no species-specific differences of the effect of cytoarchitectonic similarity on the probability of connections was observed.

The negative coefficients for the interaction between species and cytoarchitectonic similarity for the mouse–cat and mouse–macaque monkey analyses were significant. This indicates that the impact of the decrease of the cytoarchitectonic similarity on the decrease of the probability of the existence of a connection is higher in the cat and macaque monkey when compared to the mouse (see also the Materials and Methods section). For a better understanding of this effect, we visualized the impact of cytoarchitectonic similarity on the probability of the existence of a connection for different physical distance values for all pair-wise species analyses (Fig 3). For an equal decrease of cytoarchitectonic similarity, the probability of the existence of a connection decreased more slowly for the mouse when compared to the cat and macaque monkey (Fig 3).

A control analysis using the qualitative cytoarchitectonic status of the macaque monkey cortical areas and a different dataset for the mouse cortico-cortical connectivity [21] led to the same qualitative results. Specifically, the coefficients for the mouse versus cat analysis were distance = −2.21, cytoarchitectonic similarity = −0.84, and species by cytoarchitectonic similarity = −1.87 (all *p* < 0.001). The inclusion of the interaction of species and cytoarchitectonic similarity significantly improved the model fit, as indicated by an LR test (LR = 19.49, *p* < 0.001). For the mouse versus macaque monkey analysis, the coefficients were distance = −3.21, cytoarchitectonic similarity = −0.77, and species by cytoarchitectonic similarity = −2.07 (all *p* < 0.01). Also in this case, the inclusion of the interaction of species by cytoarchitectonic similarity significantly improved the model fit, as indicated by an LR test (LR = 19.23, *p* < 0.001). Finally, for the cat versus macaque monkey analysis, the coefficients were distance = −3.27 and cytoarchitectonic similarity = −2.83 (both *p* < 0.001), but the interaction of the species and cytoarchitectonic similarity regressors was not significant (species by cytoarchitectonic similarity = 0.01, *p* > 0.1). A visual depiction of the different effect of cytoarchitectonic similarity on the existence of connections in the mouse compared to the cat and macaque monkey for this control analysis is provided in (S4 Fig).

In summary, cytoarchitectonic similarity relates to the existence of connections in mammalian cortices in a species-specific manner, differentiating the mouse and marmoset monkey from the cat and macaque monkey.

### Cytoarchitectonic gradients as a central dimension related to the laminar origin of connections

**Fig 4:**
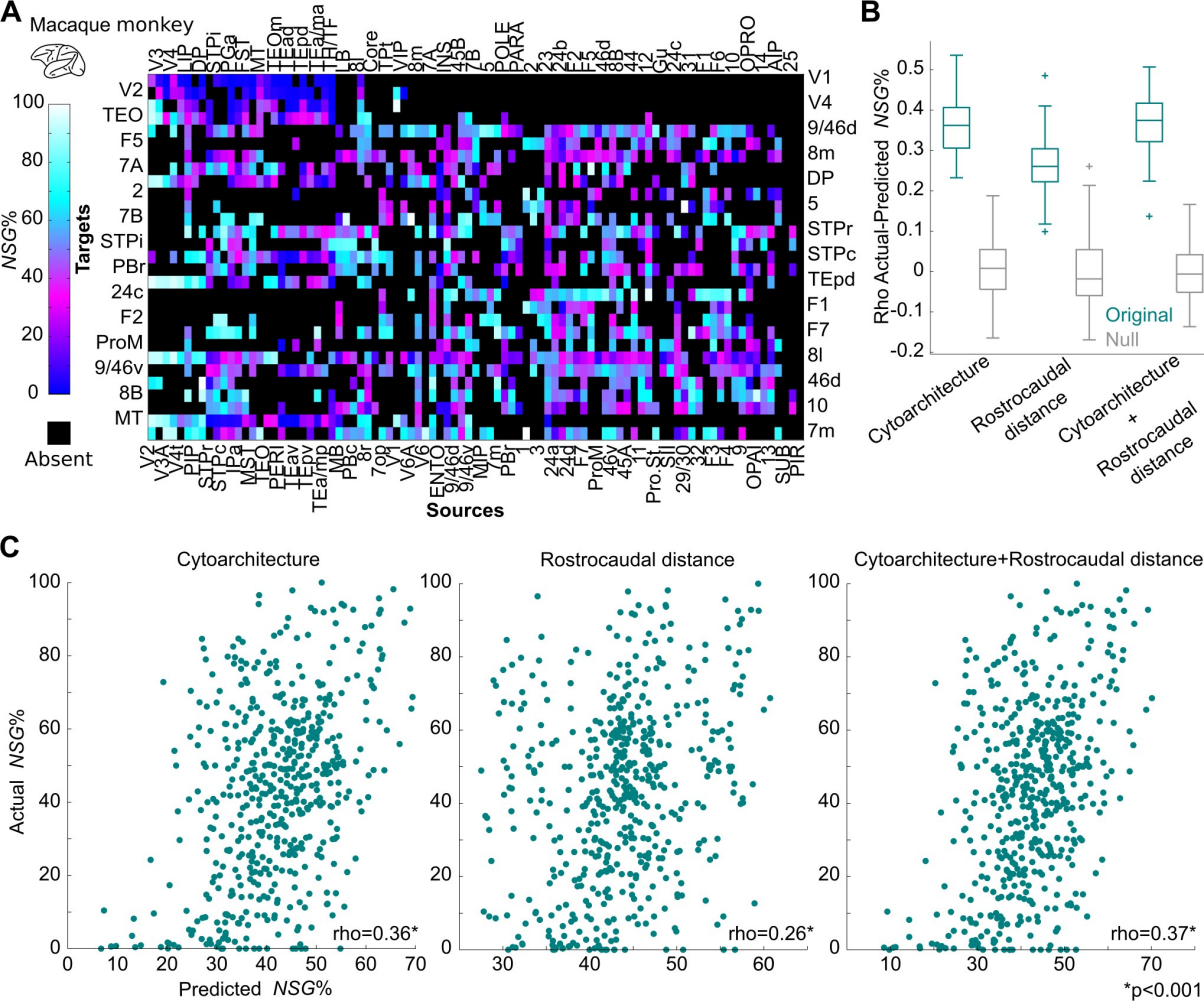
Predictions of laminar origin of connections in the macaque monkey. (A) Quantitative data of laminar origin of connections (NSG%) across areas of the macaque monkey cortex [45]. (B) Predictions of NSG% based on cytoarchitecture, distance of areas along the rostrocaudal axis, and the combination of these predictors. All predictions were statistically significant. Cytoarchitecture-based predictions led to higher correlation between actual and predicted NSG% values compared to rostrocaudal distance–based predictions (*p* < 0.001, permutation test). A combination of cytoarchitecture and rostrocaudal distance did not lead to a higher correlation between actual and predicted NSG% values compared to the use of cytoarchitecture alone (*p* > 0.1, permutation test). Thus, rostrocaudal distance did not carry additional information on NSG% values. Boxplot edges, gray lines, and whiskers and crosses depict, the 25th and 75th percentiles, median, and extreme nonoutlier and outlier values, respectively. (C) Scatterplots of actual and predicted NSG% values based on cytoarchitecture, rostrocaudal distance of areas, and the combination of these predictors. For visualization purposes, predicted NSG% values are averaged across 100 predictions. See S1 Table for full names of the cortical areas. NSG%, percentage of supragranular labeled neurons.

Next, we aimed at deciphering the central dimension of cortical organization related to the graded shifts of the laminar origin of connections across the different cortical areas. This analysis focused on the cat and macaque monkey, for which quantitative data on the laminar origin of connections were available [45, 46]. A cytoarchitecture-based model was used, based on evidence from the prefrontal [15] and visual cortex [33, 39] of the macaque monkey, highlighting the cytoarchitectonic status of the interconnected areas as predictive of the laminar origin of the connections. Here, we used quantitative information—that is, the percentage of supragranular labeled neurons (NSG%) [45] (Fig 4A)—that extends beyond the visual system of the macaque monkey and encompasses the rest of the cortex. We examined the cytoarchitecture-based model [15, 43] conjointly with a rostrocaudal-based model that corresponds to suggestions that the rostrocaudal axis of the cortex is a central predictive factor of the laminar origin of connections [14, 40–42]. We used support vector regression and partial Spearman's rank correlations. The conjoint examination of the relation of the laminar origin of connections to the rostrocaudal axis and cytoarchitectonic gradients was also performed with data from the cat cortex (see Materials and Methods).

In the macaque monkey, the cytoarchitecture-based model explained significantly more variance of the NSG% values than the rostrocaudal-based model (Fig 4B and 4C). The addition of the rostrocaudal distances as a predictor to the cytoarchitecture-based model did not lead to statistically better NSG% predictions, compared to the model based solely on the cytoarchitecture of areas (*p* > 0.1) (Fig 4B and 4C). Specifically, the Spearman's rank correlation between the actual and predicted NSG% values for the cytoarchitecture-based model was rho = 0.36, for the rostrocaudal-based model rho = 0.26, and for the combination of cytoarchitecture and rostrocaudal distances rho = 0.37 (all *p* < 0.0001). The cytoarchitecture-based model explained more variance than the rostrocaudal-based model when partial Spearman's rank correlations were estimated: the correlation between NSG% and cytoarchitecture, when partialing out the rostrocaudal distances, was rho = 0.27 (*p* < 0.0001), and the correlation between NSG% and rostrocaudal distances when partialing out the cytoarchitectonic status of cortical areas was rho = 0.10 (*p* < 0.05).

The same qualitative results were obtained when using the qualitative scale for assessing the cytoarchitecture of the macaque monkey cortex (S5 Fig). Moreover, when computing partial Spearman's rank correlations for this control analysis, the cytoarchitecture-based model explained more variance than the rostrocaudal-based model: the correlation between NSG% and cytoarchitecture when partialing out the rostrocaudal distances was rho = 0.45 (*p* < 0.0001), and the correlation between NSG% and rostrocaudal distances when partialing out the cytoarchitectonic status of cortical areas was rho = 0.08 (*p* < 0.05). Moreover, recent studies advocating the importance of the rostrocaudal axis in predicting the laminar origin of connections in the macaque monkey cortex exclude the less differentiated areas of the cingulate and insular cortex [42]. Thus, we also performed the NSG% predictions while excluding these less differentiated cortical areas. This control analysis led to the same qualitative results—that is, the cytoarchitecture-based model yielded the highest NSG% predictions—and the addition of the rostrocaudal distances did not carry any additional information (S6 Fig).

**Fig 5:**
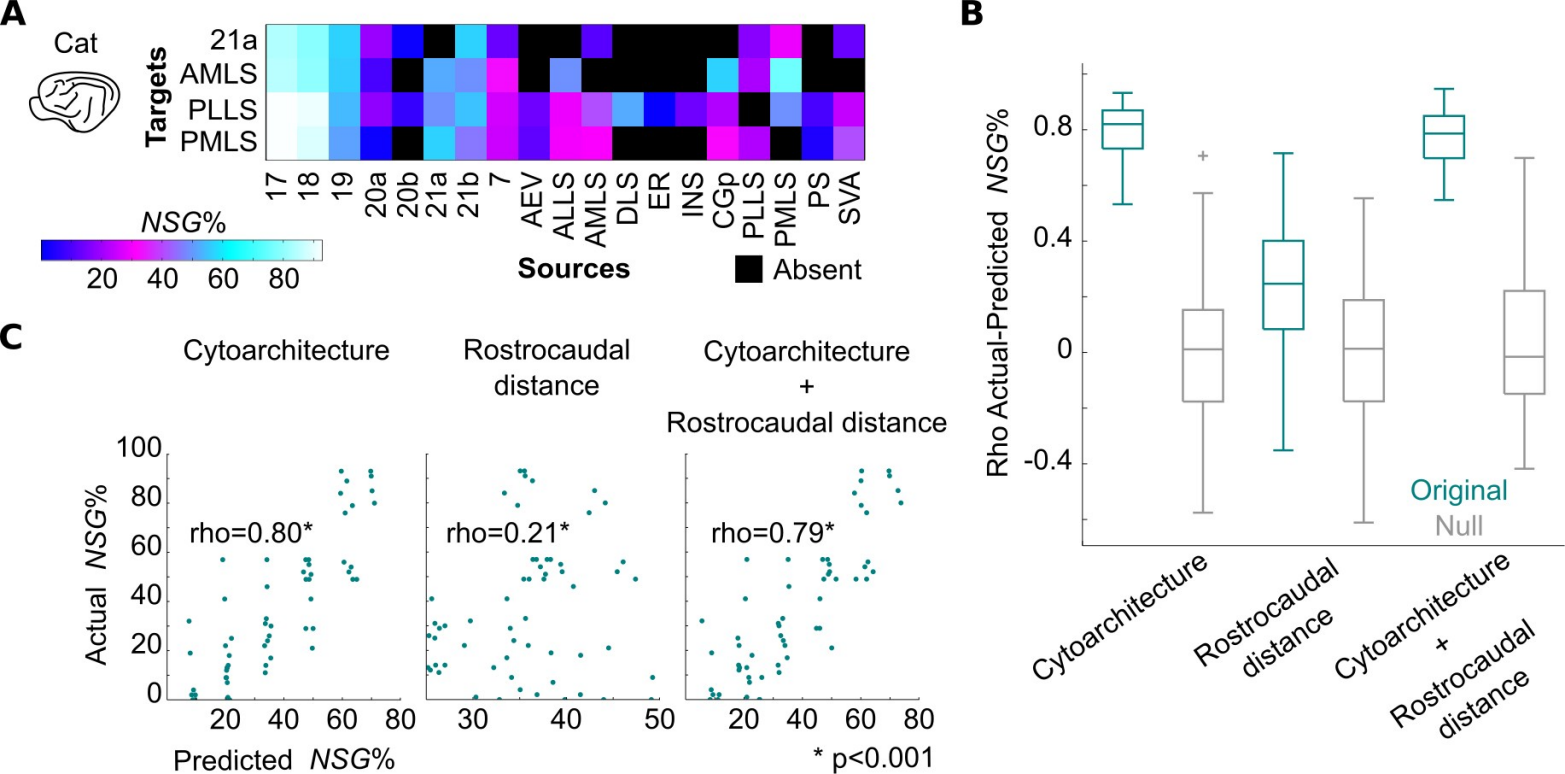
Predictions of laminar origin of connections in the cat. (A) Quantitative data of laminar origin of connections (NSG%) after retrograde injections in areas of the visual system [46]. (B) Same as in Fig 4B. (C) Same as in Fig 4C. See S1 Table for full names of the cortical areas. NSG%, percentage of supragranular labeled neurons.

The cytoarchitecture-based model also resulted in the best predictions of the NSG% values for the cat cortex (Fig 5A). The same pattern of results as for the macaque monkey cortex was observed; namely, the cytoarchitecture-based model explained significantly more variance of NSG% values when compared to the rostrocaudal-based model (Fig 5B and 5C). The addition of the rostrocaudal distances as an extra predictor to the cytoarchitecture-based model did not lead to statistically better NSG% predictions compared to the model based solely on the cytoarchitecture of areas (*p* > 0.1) (Fig 5B and 5C). Specifically, the Spearman's rank correlation between the actual and predicted NSG% values for the cytoarchitecture-based model was rho = 0.80, for the rostrocaudal-based model rho = 0.21, and for the combination of cytoarchitecture and rostrocaudal distances rho = 0.79. The same conclusions were obtained for the partial Spearman's rank correlations: the correlation between NSG% and cytoarchitecture when partialing out the rostrocaudal distances was rho = 0.79 (*p* < 0.0001), and the correlation between NSG% and rostrocaudal distances when partialing out the cytoarchitectonic status of cortical areas was rho = 0.05 (*p* > 0.1). These conclusions are further supported by an analysis of an additional dataset with categorical data on the laminar patterns of the connections in the cat cortex (S7 Fig).

**Fig 6:**
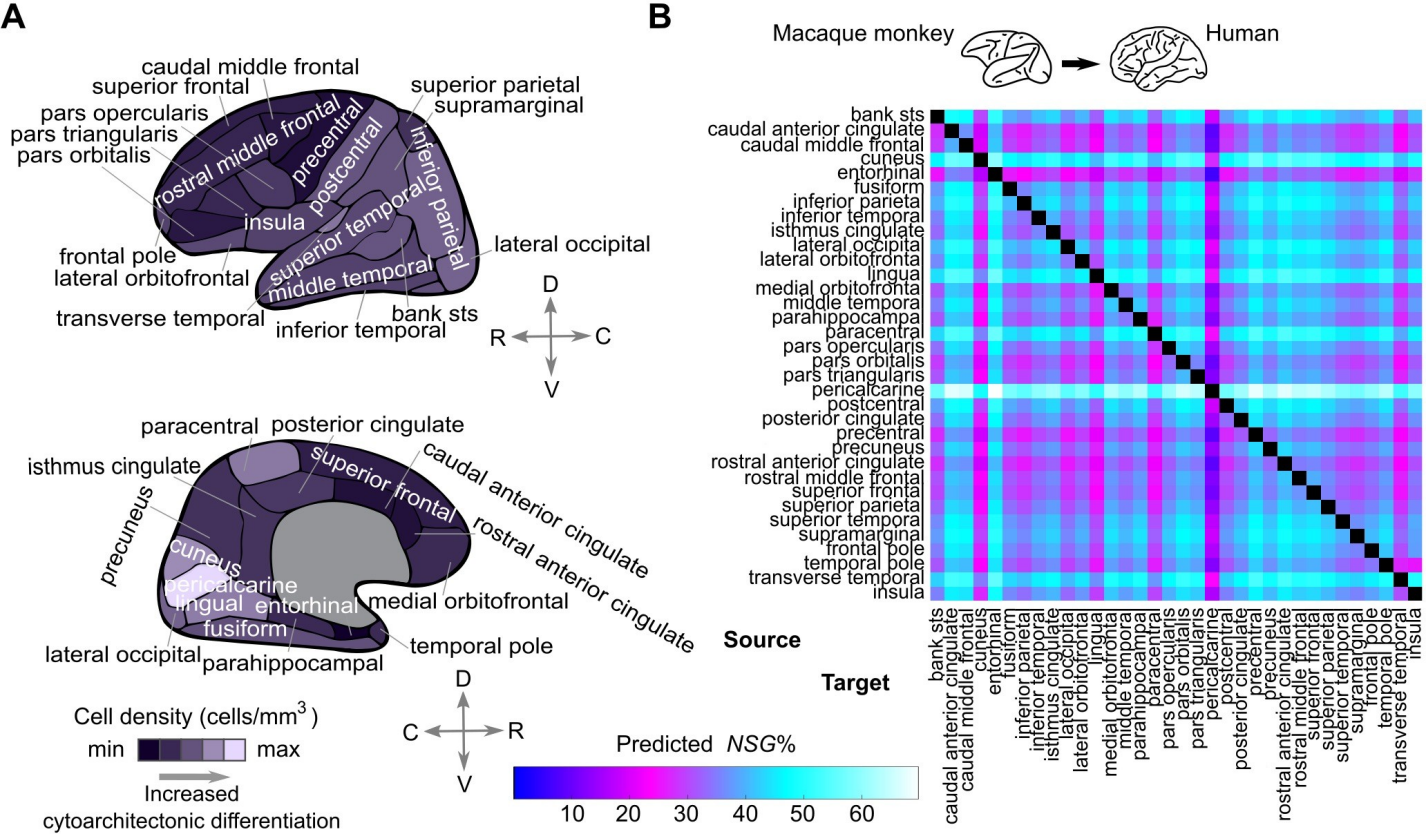
Prediction of the laminar origin of connections for the human cortex. (A) Cortical regions of the human cortex based on the Desikan-Killiany atlas [48]. Cortical regions are assigned to cortical areas S2 Table for which quantitative cell density measurements are available from the classic cytoarchitectonic map of von Economo and Koskinas [37]. The cell density of each region is the average cell density of the assigned cortical areas. (B) A cytoarchitecture-based model that was built with macaque monkey data was used to predict quantitative laminar origin values (NSG%) of putative connections in the human cortex. Such information cannot be obtained with current in vivo techniques but is essential for addressing structure–function relations in the human cortex, such as relating laminar origin of connections to interareal functional communication in different frequency channels [47]. Area-to-area NSG% predictions are not symmetric and are depicted for all pairs of areas, irrespective of the evidence for the existence of a connection in between them. C, caudal; D, dorsal; max, maximum; min, minimum; NSG%, percentage of supragranular labeled neurons; R, rostral; V, ventral.

Our results highlight the cytoarchitectonic gradients of the cerebral cortex as a central axis of organization related to the graded shifts in the laminar origin of connections across the cortical sheet. The implications of these findings are 2-fold. First, they offer a guiding thread for deciphering the cellular phenomena or mechanisms responsible for such a close systematic relation between cytoarchitecture and laminar origin of connections (see Discussion). Second, a cytoarchitecture-based model built on macaque monkey data can predict the laminar origin of connections in the human cortex, since such connectional data cannot currently be obtained by in vivo experiments (Fig 6). Such extrapolation of connectional features renders possible novel structure–function relations to be examined at a whole-cortex level, e.g., relating interareal functional communication of cortical areas and the underlying laminar origin of the connections between them [47], without the necessity of establishing macaque–human cortical area homologies.

### Mapping the structural core of the mammalian cortical network

It is known that the mouse and macaque monkey cortex possesses nonrandom topological connectivity features, such as a core–periphery structure [20, 21]. A core is a set of areas that are highly interconnected, with the remaining noncore areas constituting the periphery. A core–periphery network topology characterizes not only cortico-cortical networks but also other biological and technological networks, providing properties such as high topological efficiency [22, 49]. We aimed to map this topological structure and investigate its association with the cytoarchitectonic gradients of the cerebral cortex of the different mammals.

**Fig 7:**
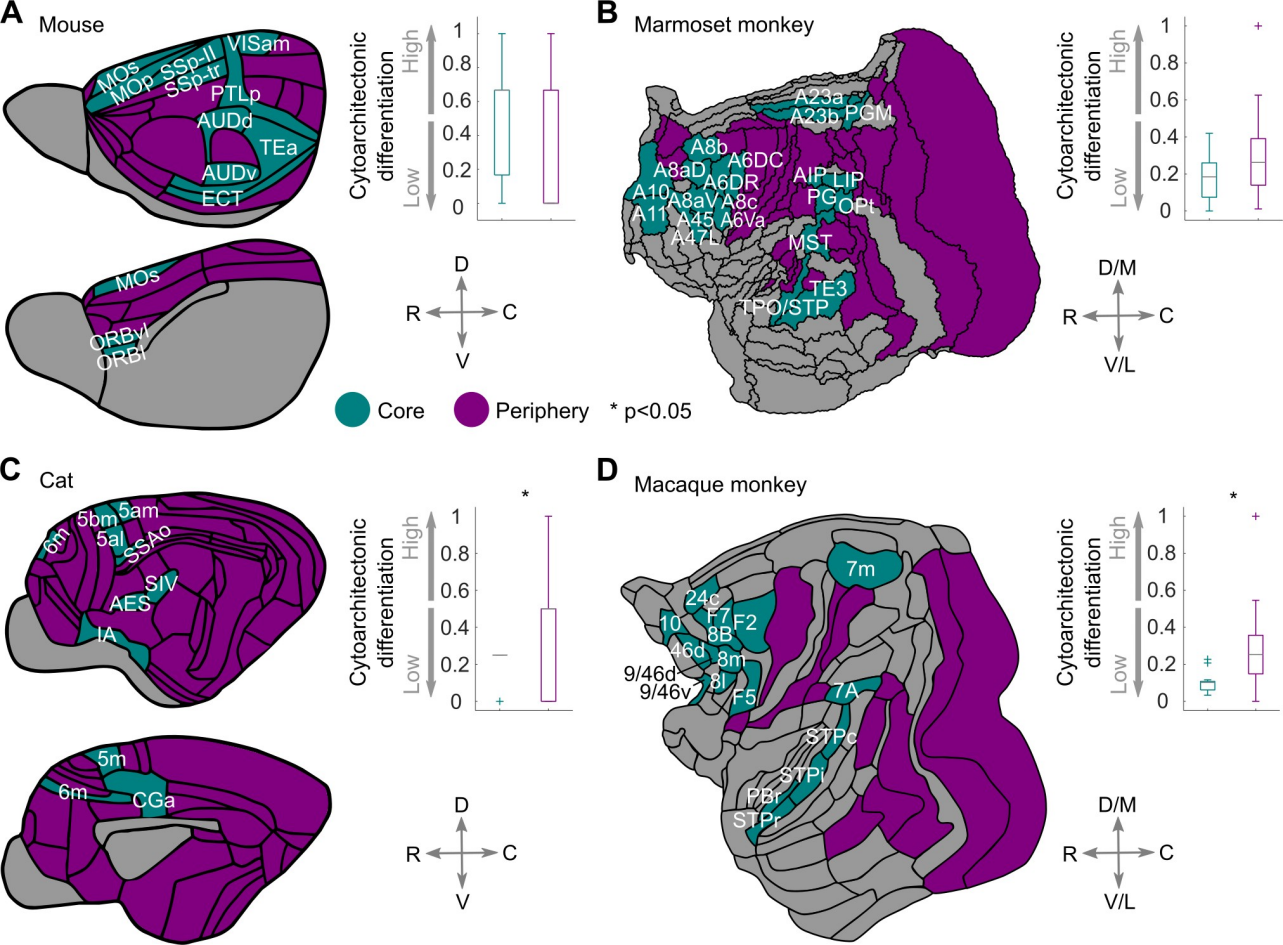
Core–periphery network topology and cytoarchitecture. The structural network core of the (A) mouse, (B) marmoset monkey, (C) cat, and (D) macaque monkey. Areas of the structural network core of the mouse and marmoset monkey do not exhibit statistically significant cytoarchitectonic differences with the areas of the periphery. In contrast, in the cat and macaque monkey, areas of the core differ significantly from areas of the periphery, with core areas exhibiting lower cortical types and neuronal density, compared to periphery areas. Boxplot edges, gray lines, and whiskers and crosses depict the 25th and 75th percentiles, median, and extreme nonoutlier and outlier values, respectively. Differences of the distributions of the core and periphery values were assessed with the Kolmogorov-Smirnov or the statistical energy test, and statistical significance was assessed with permutation tests. Note that areas colored in gray were not part of the core–periphery analyses because of a lack of data. For visualization purposes, the cytoarchitectonic status of cortical areas (cortical type or neuronal density) was linearly rescaled to the 0–1 interval. For the mouse and macaque monkey core, see also [20, 21]. See S1 Table for full names of the cortical areas. C, caudal; D, dorsal; D/M, dorsal/medial; R, rostral; V, ventral; V/L, ventral/lateral.

The core–periphery structure has already been mapped in the mouse and macaque monkey cortex by uncovering the largest cliques of the cortical connectome and forming the core as the union of areas participating in these cliques [20, 21] (Fig 7A and 7D). Here, to enable a comparative examination, we mapped the core–periphery structure with the same method in the cat and marmoset monkey. We found that the cat exhibits a core that consists of the union of two cliques of size 9 (S3 Table), whereas the marmoset monkey core is composed of the union of 12 cliques of size 16 (S4 Table). For both the cat and marmoset monkey, the size of the largest cliques forming the core was significantly different from the size of the largest cliques observed in random networks matched for degree distribution, number of nodes, and number of edges (*p* < 0.001 for both the cat and marmoset monkey, 1,000 null networks). The cat core consists of areas that have a visuomotor and multisensory integration functional signature [3] (Fig 7C). The marmoset core consists of “association” and multimodal areas of the frontal, parietal, and temporal lobe (Fig 7B).

We next related the core–periphery topology with the cytoarchitecture of the cerebral cortex. These analyses distinguished the mouse and marmoset monkey from the cat and macaque monkey. Specifically, in the mouse and marmoset monkey, the core areas did not significantly differ from the periphery areas in terms of cytoarchitectonic differentiation (Fig 7A and 7B). On the contrary, in the cat and macaque monkey cortex, significant differences were observed between the core and periphery areas, with core areas exhibiting lower neuronal densities and degree of cytoarchitectonic differentiation than the periphery areas (Fig 7C and 7D). Thus, although a structural network core characterizes the cortico-cortical network of all examined species, this topological structure is related differently to the cytology of the cerebral cortex, with a shift of the network core to less neuronally dense and differentiated areas of the cortex as the arbiter between the examined species.

**Fig 8:**
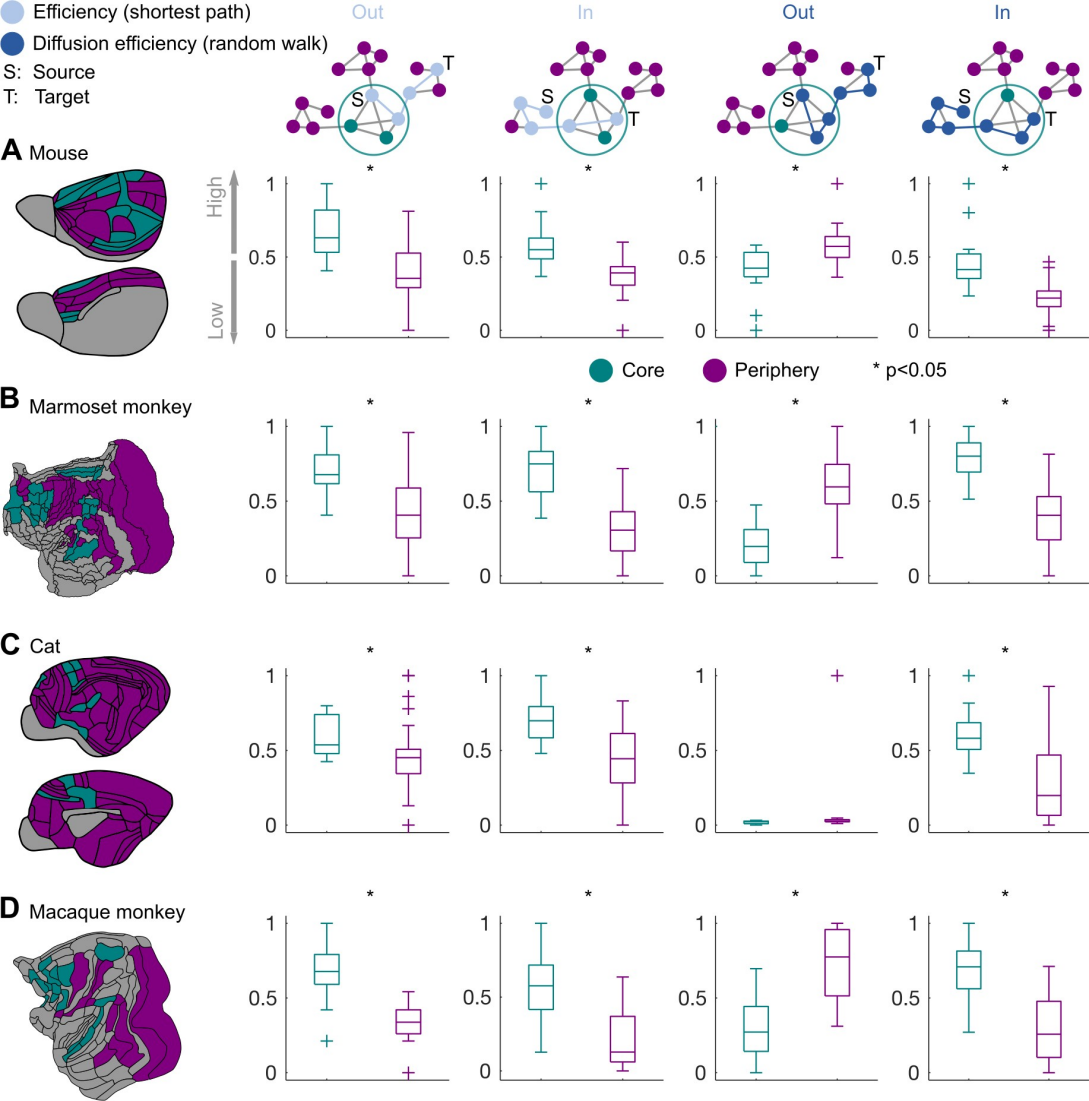
Core–periphery topology and network efficiency. Core areas of the (A) mouse, (B) marmoset monkey, (C) cat, and (D) macaque monkey connectome exhibit higher incoming efficiency than the periphery areas. Higher incoming efficiency is observed for the core under two different modes of network communication—that is, when a shortest path (efficiency) or random walk (diffusion efficiency) mode of communication is assumed. Thus, under both modes of network communication, core areas can be reached faster than the periphery areas. The core areas also exhibit higher outgoing efficiency for the shortest path, but not for the random walk, mode of communication. Thus, for fast communication with other cortical areas, the structural core must adhere to a mode of communication that is geared toward shortest paths. Boxplot edges, lines, and whiskers and crosses depict the 25th and 75th percentiles, median, and extreme nonoutlier and outlier values, respectively. Differences of the distribution of efficiency values for the core and periphery areas were assessed with the Kolmogorov-Smirnov test, and statistical significance was assessed with permutation tests. Note that for visualization purposes, efficiency values were linearly rescaled to the 0–1 interval.

We subsequently proceeded to the explicit elucidation of the role of the network core in the communication among cortical areas. To this end, we examined the efficiency of the core areas, under two diametrically opposite and recently suggested scenarios of network communication [50]. Specifically, we assessed if the core exhibited higher efficiency under the scenario that communication in the cortical network takes place via the shortest paths or as passive diffusion (corresponding to random walks) [50]. For all species and both modes of communication (shortest path or random walk), core areas exhibited higher incoming efficiency than the periphery areas (Fig 8). Thus, the core, compared to the periphery, can be reached faster from cortical areas under both modes of communication. Moreover, for all species, the core areas also exhibited higher outgoing efficiency for shortest paths but not for the random walk mode of communication (Fig 8). Thus, under the random walk mode of communication, core areas, compared to the periphery, are not topologically privileged for fast access to other cortical areas. Hence, in the context of the two aforementioned modes of network communication, the structural core must adhere to a mode of communication geared toward shortest paths in order to achieve fast access to the areas of the cortical network.

In sum, the structural core, which is central for network communication, constitutes a common network topology of diverse mammals. The core–periphery topology is related to the cytology of the cortex in a species-specific manner. Specifically, a displacement takes place toward the less differentiated and overall neuronally dense areas of the cerebral cortex when transitioning from the mouse and marmoset monkey to the cat and macaque monkey.

## Discussion

**Fig 9:**
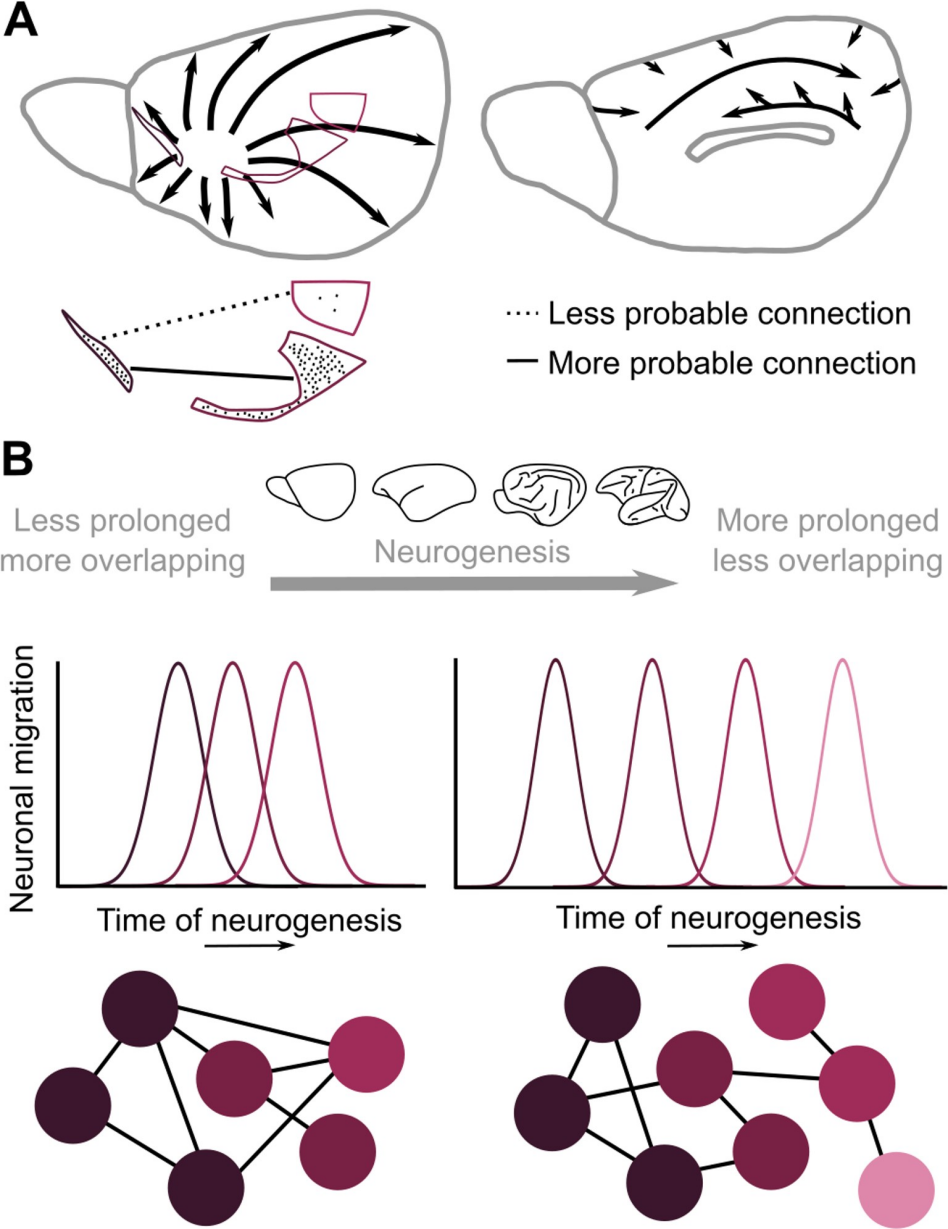
Putative neurodevelopmental mechanisms underlying the observed preferential connectivity between cytoarchitectonically similar cortical areas. (A) The cytoarchitecture of areas in the adult cerebral cortex might reflect their distinct time courses in neurogenesis. Heterochronous and spatially ordered neurogenetic gradients indicate distinct time windows in neurogenesis in the mouse, with arrows denoting the direction of propagation of neuron release and accumulation [53]. Hence, similar cytoarchitecture might entail a similar time course of neurogenesis, thus biasing the cortical connections to form primarily between areas with similar overlapping time windows, since they host more neurons functioning as probable “connection partners.” (B) The duration of neurogenesis is shorter in mice compared to macaque monkeys [54]. Overall, a shorter neurogenetic period and less distinct time windows of neurogenesis may result in the observed species-specific relation of the existence of connections and the cytoarchitecture of the cerebral cortex.

**Fig 10:**
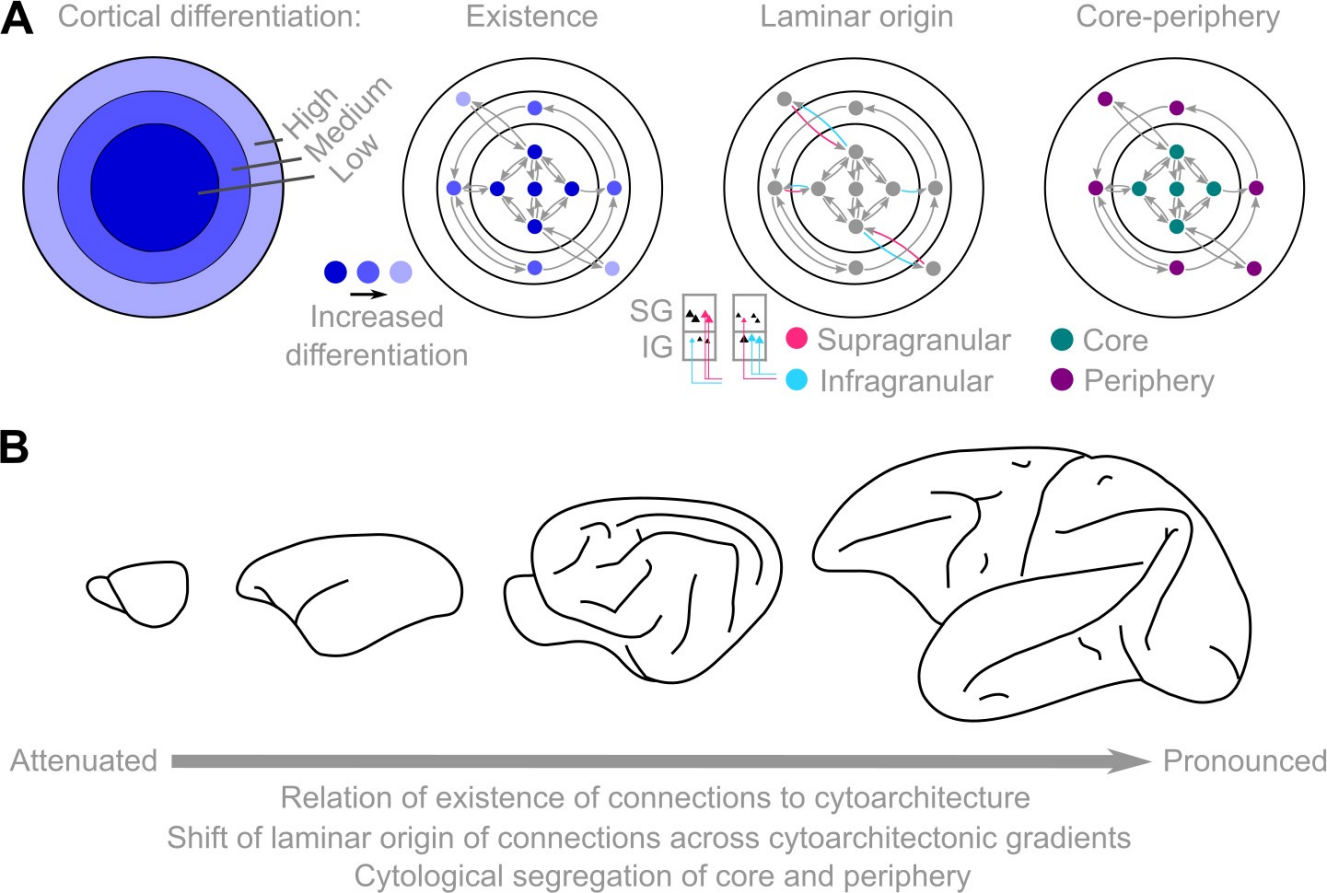
Unifying principles of mammalian connectomes and their common and diverse manifestation across species. (A) Cytoarchitectonic gradients of the cerebral cortex and their relation to fundamental interareal connectome features (existence and laminar origin of connections) and global network topology (core–periphery). (B) The relation of cytoarchitecture and connectome features is manifested in a species-specific manner, distinguishing the mouse and the marmoset monkey from the cat and macaque monkey. The direction of the arrow denotes pronounced correspondence of cytoarchitectonic similarity and existence of connections, pronounced shifts of the laminar origin of connections across the cortical sheet, and neuronal sparsification of the structural core, resulting in the segregation of the cytology of core and periphery areas. IG, infragranular; SG, supragranular.

The present results reveal unifying principles that relate interareal and global network topology properties of cortical connectomes with the physical and cytoarchitectonic dimension of the cerebral cortex, thus extending previous comparative connectome studies [2, 9, 51, 52]. These principles are manifested in a species-general but also in a systematic species-specific manner that distinguishes the smaller mouse and marmoset monkey cortex from the larger cat and macaque monkey cortex. Specifically, the existence of connections is related to the cytoarchitectonic similarity of cortical areas, above and beyond the role of physical distance, in cats and macaque monkeys but is attenuated or even absent in mice and marmoset monkeys. This relation may reflect modifications of evolutionarily conserved developmental mechanisms (Fig 9). The cytoarchitectonic status of cortical areas, and not their physical embedding across the rostrocaudal axis, is more closely linked to the laminar origin of connections, allowing the extrapolation of this connectional feature to humans. Lastly, a network core characterizes all cortical connectomes, with a displacement of the core toward the less differentiated and overall neuronally dense areas of the cerebral cortex in cats and macaque monkeys (Fig 10). In sum, our results sketch out a blueprint of mammalian connectomes by highlighting the species-specific and species-general links between the connectional, physical, and cytological dimensions of the cerebral cortex.

### Wiring cost, cytoarchitectonic gradients, and cortical connections

The premise that neuronal systems are wired in such a way that minimizes the physical distance between the interconnected elements explains part of the characteristic pattern of presence and absence of connections between cortical areas in different mammalian species [9, 20, 21, 29–31]. The current examination demonstrated that wiring cost also constrains the cortical connectome of a New World monkey—that is, the marmoset monkey.

The current comparative framework allowed us to gain deeper insights into how the relation of cytoarchitectonic similarity to the existence of connections manifests across species. Our results reveal that cytoarchitectonic similarity has an attenuated impact on the probability of the existence of connections in the mouse when compared to the cat and macaque monkey and is statistically absent in the marmoset monkey. Thus, unifying wiring principles linking connectivity and cytoarchitecture not only distinguish rodents from primates but also point out differences within the primate order. Primates are distinguished from rodents with respect to the scaling of the size of the brain and the number of neurons it contains [55]. Our study offers further comparative insights by relating the cytology of the cortex to its macroscale connectivity and assessing how this relation is manifested in different mammals. The species-specific manifestation of the relation of connectivity and cytoarchitecture is systematic and highlights the trajectory of this relation across the mammalian spectrum. Specifically, our framework predicts that, on average, the relation of cytoarchitectonic similarity and the existence of connections in smaller mammalian cortices (e.g., hamster, treeshrew) might be attenuated or even absent when compared to larger mammalian cortices (e.g., great apes, humans). Modifications of evolutionarily conserved developmental mechanisms may be the cause for this species-specific relation of cytology and connectivity (Fig 9) (see “Heterochronous, graded neurogenesis and pyramidal cell size heterogeneity”). The current results complement and resonate well with recent findings that show a common, but also species-specific manifestation, of the role of physical distance in the wiring of the mouse and macaque monkey cortex [21].

The current quantitative cross-species examination also allows to decipher the central cortical dimension that relates to the graded shifts of the laminar origin of cortico-cortical connections. In both the cat and macaque monkey, the laminar origin of the connections is dictated by the cytoarchitectonic status of the interconnected areas and not the orientation of a connection along the rostrocaudal spatial axis. Thus, the current investigation, conjointly with previous results [33, 39, 56], highlight the close relation of cortical cytoarchitectonic gradients to the systematic shifts of the laminar origin of connections. The demonstration that cytoarchitectonic differentiation is a central cortical dimension related to the laminar origin of connections offers the ground for deciphering the concrete cellular phenomena across the cortical sheet that might be responsible for the shifts of the laminar origin of cortico-cortical connections (see “Heterochronous, graded neurogenesis and pyramidal cell size heterogeneity”). In addition, the close relation of cytoarchitectonic gradients and laminar origin of connections can be used for extrapolating this connectional feature to the human cortex (see “Unifying wiring principles allow cross-species predictions”). We should also note that the cytoarchitectonic gradients of the cerebral cortex explain a substantial part of the variance of the shifts of the laminar origin of connections, but not the total variance; thus, it is important to uncover additional factors that may shape the laminar shifts of connections across the cortical sheet of mammals.

### Unifying wiring principles allow cross-species predictions

The species-general finding that cytoarchitectonic gradients constitute a central cortical dimension related to the laminar origin of connections not only offers neurobiological insights but also allows the extrapolation of this connectional feature from macaque monkeys, the closest primate to humans that can be invasively examined, to the human cerebral cortex. Such extrapolation allows novel structure–function examinations. For instance, frequency-dependent communication between areas of the human visual system obeys the same structure–function principles observed in the macaque monkey [12, 47]. Our results allow extrapolation of the laminar origin of connections to humans, thus allowing such structure–function examinations to be performed at the global, whole-cortex level without the need for establishing homologies between the cortical areas of the two species. In sum, our results, conjointly with recent efforts [57, 58], demonstrate the value of uncovering unifying principles that can be used to link a wealth of data on the macaque monkey cerebral cortex to the human cerebral cortex.

### Displacement of the structural network core in mammals

Our results highlight a structural network core in the mammalian cerebral cortex, important for the communication of cortical areas. Our comparative analysis demonstrates the displacement of the structural network core in the mammalian phylogeny. This displacement is manifested as a species-specific relation of the network core to the cytology of the cortex, leading to the neuronal sparsification of the core in cats and macaque monkeys—that is, the displacement of the network core toward the least neuronally dense parts of the cerebral cortex.

We have demonstrated that across mammalian species, the structural core, compared to the periphery, is reached faster from cortical areas under two modes of network communication—that is, communication based on passive diffusion or shortest paths [50]. Passive diffusion is not costly from an information point of view, since navigating the network relies on random transitions from area to area, with no “knowledge” about the topology of the network. Shortest paths, on the other hand, require the channeling of communication between cortical areas through the shortest routes of the cortical network, and thus this mode of communication is considered information-costly [50]. Passive diffusion traps signals inside the densely interconnected core; hence, the structural core can reach faster, compared to the periphery, other areas only under the adoption of a more information-costly mode of communication that is geared toward shortest paths. Topologically central parts of the brain also exhibit a high energetic cost [59, 60], and thus a substantial part of this energetic cost, or overall energy consumption of the mammalian brain, might be attributable to the need of the areas of the core to adopt an information-costly mode of communication in order to achieve fast communication with other cortical areas. In sum, we highlight the importance of the structural network core in the communication of cortical areas. These findings provide an empirical foundation to theoretical frameworks [22] and extend previous results [9] by situating the structural network core in a comparative context and elucidating its role in light of recently suggested taxonomies of network communication processes.

The displacement of the network core across the cortical gradients, leading to its neuronal sparsification in cats and macaque monkeys, highlights three points with potential functional ramifications and suggests varied degrees of vulnerability to pathologies.

First, less cytoarchitectonically differentiated—and thus overall less neuronally dense—areas in mammalian cortices, such as the areas of the insular and cingulate cortex, are also overall less myelinated when compared to more differentiated areas, such as primary sensory-motor areas [35, 61–63]. Both in vivo and in vitro studies in mammals demonstrate that high degree of myelination suppresses synaptic plasticity and axonal growth [64–66]. Thus, less myelinated areas are more flexible than more myelinated areas [67]. Therefore, the cat and macaque monkey network core, in contradistinction to the core of the mouse and, to a certain extent, marmoset monkey, seems to include the most-flexible areas of the cerebral cortex, bestowing the network core in these species with higher degrees of adaptability.

Second, in the macaque monkey, spine densities vary across cortical areas, with less differentiated and overall neuronally dense areas exhibiting high spine densities. In mice, differences of spine densities across cortical areas are very attenuated [68, 69]. Computational modeling employing the heterogeneity of spine densities across areas as a proxy for the strength of excitatory input to pyramidal cells demonstrates that spine density heterogeneity is important for the generation of temporal receptive windows across cortical areas, bestowing the less differentiated parts of the cortex with more-prolonged time windows that are ideal for integration of signals over longer time periods [45]. Thus, the above computational evidence and interspecies differences with respect to spine density heterogeneity across the cortical gradients indicate that in macaque monkeys, contrary to mice, the alignment of the network core with areas exhibiting high spine densities constitutes a synergy of connectional and microcytological features that may enhance the functional integration capacity of the core in macaque monkeys.

Third, although the neuronal sparsification of the network core might entail differences in terms of integration and plasticity, it might also entail an increased vulnerability. Less neuronally dense areas also exhibit high metabolism and cellular stress [67]. Highly connected areas, like the areas of the network core that we have currently highlighted, are more affected in diverse pathologies [70]. Thus, a cortex characterized by a synergy between highly connected and neuronally sparse areas can also entail an increased vulnerability to pathologies.

In sum, our results highlight the relation of the network core to the cytology of the cortex across different mammals and the functional ramifications of such relation, thus situating the topology of the mammalian connectome in a comparative and neurobiologically interpretable context.

### Heterochronous, graded neurogenesis and pyramidal cell size heterogeneity

Uncovering unifying wiring principles of the cerebral cortex harnesses the complexity of cortical wiring and renders possible a glimpse into the neurodevelopmental mechanisms suggested by these principles. The more pronounced relation of cytoarchitectonic similarity to the existence of connections observed in cats and macaque monkeys in relation to mice and marmoset monkeys may be rooted in the spatiotemporal structure of neurogenetic gradients during development [16, 36, 53, 71, 72]. Specifically, the spatially ordered cytoarchitecture of cortical areas might reflect the spatially ordered heterochronous neurogenesis and subsequent migration of neurons across the developing pallium. Hence, areas with similar cytoarchitecture might also exhibit similar developmental time courses [27, 36, 73]. Therefore, areas with similar cytoarchitecture in the adult cortex might be more likely to be connected, since during development they host neurons that constitute more-readily available connection partners, following a “what develops together, wires together” principle [39]. This mechanistic explanation assigns a central role to the heterochronicity of neurodevelopmental events in the formation of intricate wiring configurations. Such a mechanism is directly supported by empirical studies investigating the neurogenesis and connections of the olfactory bulb and the primary olfactory cortex in rats [72] and computational modeling [74, 75]. The duration of neurogenesis is shorter in mice compared to macaque monkeys [76] and possibly arises from a common evolutionarily conserved mechanism [54]. Less distinct time windows and overall shorter neurogenesis in the mouse and the marmoset monkey, when compared to the cat and macaque monkey, may result in the currently observed species-specific manifestation of the relation of existence of connections and the cytological composition of cortical areas (Fig 9).

In addition, computational modeling [77] and empirical evidence from *Caenorhabditis elegans* suggest that heterochronicity in neurogenesis might also partially explain the formation of a structural core; that is, neurons that constitute a tightly interconnected core in the adult worm are born earlier than noncore neurons [78]. A similar “early neurogenesis advantage”, in addition to spatial constraints imposed by the geometry of the cortex [20] or the distinct molecular signature of core areas [79], might constitute factors that lead to the formation of a network core in the mammalian cortex.

Our results demonstrate a tight relation of laminar origin of connections and cytoarchitecture. Why do less differentiated cortical areas elicit connections predominantly from infragranular layers, whereas more-differentiated areas elicit connections from predominantly supragranular layers? Part of the answer might lie in the phenomenon of externopyramidization (*Externopyramidisierung*) [38, 80, 81]. This structural organization principle of the cerebral cortex, observed in diverse species, including humans [38, 61, 80, 82], describes the rate of change of the ratio of the soma size of pyramidal neurons located in upper (supragranular) layers versus lower (infragranular) layers across the cortical sheet. In mammalian cortices (for instance, cat and monkey cortices), the progressive differentiation of areas is accompanied by an increase of the soma size of supragranular pyramidal cells relative to the soma size of the infragranular pyramidal cells [81]. A larger soma size of pyramidal cells entails larger axon diameters, higher conduction velocities, and larger boutons that contain a higher number of vesicles, leading to higher probabilities and larger amounts of neurotransmitter release [81]. Therefore, the soma size of pyramidal neurons entails ultrastructural and functional properties of the corresponding axons, possibly rendering connections originating from pyramidal neurons with large soma more suitable for high-throughput long-range communication. Consequently, the phenomenon of externopyramidization might partially explain why less differentiated areas can establish and maintain long-distant connections primarily from infragranular layers, whereas more differentiated areas primarily from supragranular layers [81]. The phenomenon of externopyramidization is manifested with a varied degree of prominence across the mammalian spectrum, thus allowing the prediction of the laminar origin of connections in not-yet-examined species [81]. Specifically, in species with an attenuated manifestation of the phenomenon of externopyramidization, like mice, less pronounced shifts of the laminar origin of connections across the cortical sheet will be observed, whereas in species with a more prominent manifestation of the phenomenon of externopyramidization, like gorillas and humans, more pronounced shifts of the laminar origin of connections will be observed, with a gradual emphasis on supragranular layers [81].

In sum, our results highlight specific neurogenetic and cellular phenomena giving rise to unifying principles linking the cytoarchitectonic and connectional organization of the adult mammalian cortex.

### Future directions

Gradients of cortical differentiation entail changes of multiple cortical features, such as myelin and density of different receptors and interneuron subtypes [16, 80, 83]. Thus, apart from obtaining more comprehensive quantitative cytoarchitectonic data, future studies in mammals should also elucidate how macroscale connectivity relates to other dimensions of cortical architecture. Moreover, changes across cortical gradients are layer-specific [27]. Therefore, in order to reveal a more fine-grained picture of cortical architecture, quantitative measurements should ideally also be obtained in a layer-wise manner. Furthermore, additional features such as the strength heterogeneity of connections, as well as new results from invasive tract-tracing studies [84], should be examined. In the macaque monkey, connectivity strength heterogeneity is related not only to the physical embedding of the cortex but also to the homophily principle—that is, the connectional similarity of cortical areas [85]. Thus, we predict that the homophily principle will help explain the strength heterogeneity of cortico-cortical connections in other mammals. Lastly, in phylogenetically close species, such as monkeys and humans, common long-range fiber systems can be discerned and used for the examination of species-general and species-specific organizational principles [86]. Such an approach, in conjunction with the approach that we have adopted, increases the tools for quantitative cross-species examinations and hopefully will further pave the way for additional insights into the organization of mammalian cortices.

### Conclusions

Our results sketch out a connectional blueprint for the mammalian cerebral cortex by demonstrating species-general and systematic species-specific unifying principles linking the connectional, cytological, and physical dimensions of the cerebral cortex. The common principles allow the extrapolation of connectional features to not-yet-examined mammalian species, whereas the species-specific variations highlight unique aspects of cortical organization across the mammalian spectrum with potential function ramifications. Commonalities and differences of cortical organization may stem from variations and persistence of evolutionarily conserved neurodevelopmental mechanisms and cellular phenomena.

## Materials and methods

All analyses were performed in MATLAB (MATLAB 2016a, MathWorks, Natick, MA, USA). The datasets used are freely available from the indicated resources and are the result of the cited published studies. No additional animal experiments were conducted for the present study.

### Connectome datasets

#### Mouse cortex

For the mouse (*Mus musculus*), we used the data described in [7], which constitute a comprehensive connectome of the cerebral cortex. We used a dataset based on the logical AND of the cortico-cortical connections revealed from retrograde and anterograde tracers. The logical AND entails that a connection was considered present only if it was deemed present in both datasets obtained from anterograde and retrograde tracers. The mouse connectome was a 48 × 48 connectivity matrix. We also used the dataset used in [21], which is a combination of the datasets described in [6, 7] (33 × 33 connectivity matrix). Both of these datasets are compiled based on a common parcellation scheme of the mouse cortex [87]. Data on the laminar origin of the connections in the mouse cortex are not yet available at a whole-cortex level. Hence, analysis of laminar patterns did not involve the mouse. The cytoarchitecture of cortical areas of the mouse was assessed qualitatively by defining an ordinal scale of cortical types based on Nissl-stained sections. Specifically, the criterion for assigning an area to category 1 was the absence of layer IV—that is, if an area was agranular. Areas assigned to category 2 did not have a clearly discernible layer IV and hence were characterized as dysgranular. Cortical areas assigned to category 3 were characterized by the presence of layer IV and were thus granular areas. Areas assigned to category 4 were more clearly eulaminated; that is, they exhibited a more distinct differentiation of layers accompanied by a thick and dense layer IV [31]. Note that four areas—VISam, VISpm, VISl, and VISpl—exhibited an intermediate type between 2 and 3 and were thus assigned to a separate category (2.5). Physical distance was based on an approximation of axonal path lengths or the Euclidean distance between the barycenters of the cortical areas of the mouse atlas [87].

#### Cat cortex

The cat connectome was based on a meta-analysis of tract-tracing studies in the cat [3], which is the only available cat whole-cortex connectome to date. This dataset was a 63 × 63 connectivity matrix. The cytoarchitecture of areas was assessed qualitatively based on Nissl-stained sections as described in [30]. Specifically, the cat cortex was composed of areas of the highest structural differentiation (type 5), for which identifying the different layers was easy, and areas of the lowest differentiation (type 1), for which determining the layers was hardest, followed by areas in the adjacent categories (types 4 and 2), which were almost but not quite as well or as poorly differentiated as the highest and lowest types. Finally, the remaining areas, possessing an intermediate structural differentiation, were classified as type 3. The striate (area 17) and parastriate (area 18) cortices were rated as the most highly differentiated areas of cortex (cortical type 5), as they possess the widest and most densely granularized layer IV. Areas rated as the most poorly differentiated (cortical type 1) exhibited features typically observed in what have been termed the “paralimbic” cortices and are characterized by blurred and low-density cell layers (except for a cell-dense rim in the outermost part of layer II); exhibited a negligible or indistinguishable layer IV, which is very weakly granular or agranular; and also exhibited a relatively expanded layer VI [30, 46]. Physical distance was based on border distances—that is, the number of areas that need to be crossed to reach one area from another. The use of the border distance is necessitated in the absence of a 3D stereotaxic atlas of the cat cortex with the nomenclature of [3]. Quantitative data on the laminar origin of connections of the cat cortex were used from injections in four visual cortical areas [46]. These data offer a graded continuous measure of the laminar origin of cortico-cortical connections by quantifying the NSG% in a cortical area after retrograde injections [15]. For example, after injecting area A, 100 neurons may be labeled in area B, out of which 20 are located in supragranular layers and 80 in infragranular layers. Therefore, NSG% would be 20/(80 + 20) = 0.2 * 100 = 20, indicating a predominant infragranular origin for this connection. Hence, NSG% values range from 0 to 100, with values closer to 0 denoting predominantly infragranular origin and closer to 100 predominantly supragranular origin. Moreover, we used an independent dataset with a binary “feedforward” and “feedback” classification of connections based on the laminar origin and termination of connections for a subset of cortical areas [88].

#### Macaque monkey cortex

The macaque monkey (*Macaca fascicularis*, *macaca mulatta*) cortical connectome was based on [8]. The connectivity matrix was the result of retrograde injections in 29 cortical areas, resulting in a 29 × 91 connectivity matrix. Cytoarchitecture of areas was assessed qualitatively and quantitatively. Neuronal density (that is, the number of neurons per mm^3^ [33]) was used as a quantitative measure that constitutes a structural fingerprint of cortical areas related to the degree of cytoarchitectonic differentiation of an area [36]. For the neuronal density measurements, both NeuN- and Nissl-stained sections were used, with a near-perfect correlation of the two measurements for areas for which both stainings were available [33]. Moreover, qualitative assessment was performed by defining an ordinal scale of cortical types based on Nissl-stained sections. For detailed criteria and an analogous procedure, see [39]. The laminar origin of the cortico-cortical connections was based on the data described in [45], which involve retrograde injections in 29 areas and detection of the laminar position of the projection neurons in the rest of the cortex. This dataset spans different lobes of the macaque monkey cortex and thus extends previous analysis focused on the visual system [33, 39, 89]. Physical distance between areas was based on the geodesic distance, constrained by the white matter, between the barycenter of the cortical areas as described in [8,20].

#### Marmoset monkey cortex

The marmoset monkey (*Callithrix jacchus*) connectome was based on digitized invasive tract-tracing injections involving 55 cortical areas, resulting in a 55 × 115 connectivity matrix (see [10] for details and [90] for the parcellation scheme). Cytoarchitecture of areas was quantitatively assessed based on NeuN-stained sections (see [44] for details). Physical distance between areas was computed as the Euclidean distance between the barycenters of the cortical areas described in [90].

### Prediction of connection probabilities

We used binary logistic regression for the prediction of the existence of connections across species. In order to render cross-species predictions feasible, the predictors (physical distance and cytoarchitectonic similarity) were linearly normalized to the 0–1 interval separately for each species. Note that this normalization does not artificially expand or shrink the levels of differentiation or size of each species, since the relative changes indicated by these regressors are of importance. Subsequently, a model was built with the existence of connections as a binary dependent variable and the physical distance and cytoarchitectonic similarity as predictors. We were interested in investigating if the cytoarchitectonic similarity of cortical areas relates to the existence of connections in a species-specific manner. Therefore, a categorical predictor coding for the different species was added to the model as well as the interaction of this predictor and the cytoarchitectonic similarity predictor. The improvement of the model fit, when the interaction of species and cytoarchitectonic similarity was included, was assessed with the LR test.

### Predicting the laminar origin of connections

For predicting the laminar origin of connections, an out-of-sample classification approach was adopted. We used support vector regression with a regularization parameter C = 1. A cytoarchitecture-based model, quantifying the difference of the cytoarchitecture of the cortical area of projection origin versus the cytoarchitecture of the cortical area of projection termination, was built on 70% of the data and tested on the remaining data. The predictions were computed 1,000 times, each time using 70% of the available data to build the model (drawing without replacement). The quality of the predictions was assessed by computing the Spearman's rank correlation between actual and predicted NSG% values. In the same fashion, a rostrocaudal-based model was built and tested. The coordinates of the barycenters of the cortical areas along the rostrocaudal axis were normalized to the 0–1 interval, with 0 denoting the most caudal area and 1 the most rostral area. Subsequently, for each connection the rostrocaudal coordinate of the connection origin was subtracted from the rostrocaudal coordinate of the connection termination. Hence, increasingly positive (negative) values of this rostrocaudal distance metric denote increasing rostral-to-caudal (caudal-to-rostral) distances. A 3D stereotaxic atlas was used for the macaque monkey [8]. For the cat cortex, in the absence of a 3D stereotaxic atlas, we used the 2D atlas of Scannell and colleagues [3]. The map was digitally reproduced, each cortical area was color-coded with a unique color, and the map was imported in MATLAB. Each area was assigned to a position along the rostrocaudal axis in this native coordinate system by computing the mean coordinate in the y-axis of all the pixels belonging to each area, and subsequently rostrocaudal distances were computed as described for the macaque monkey cortex. For estimating the unique variance explained by each predictor, we additionally computed partial Spearman's rank correlations between the NSG% values and the rostrocaudal distance and the cytoarchitectonic difference of the connection origin and termination. For the cat cortex, the laminar origins of the connections were available either as quantitative NSG% values or as a binary category—that is, “feedforward” or “feedback.” For the NSG% values, the Spearman's rank correlation between the predicted and actual NSG% values was used for assessing the quality of the predictions. For the binary case, the quality of the predictions was assessed by computing the corresponding area under the curve of the receiver operating characteristic curves. Null predictions and significance levels were obtained by training the model 100 times on shuffled NSG% values or binary labels.

### Detecting the core–periphery structure

For detecting the core in the cortico-cortical network, we followed the approach described in [20]. We used a MATLAB implementation of the Bron-Kerbosch algorithm with pivoting and degeneracy ordering (https://de.mathworks.com/matlabcentral/fileexchange/47524-find-maximal-cliques-for-large—sparse-network) to detect the largest cliques (that is, sets of fully connected areas). The core was defined as the union of areas participating in the largest cliques, and the rest of the areas were assigned to the periphery. Applying this algorithm to the macaque monkey and mouse data resulted in the exact same core areas as the ones reported in [20] and [21]. We applied the same algorithm for detecting the core areas of the cat and marmoset monkey connectome. To test if the core observed in the empirical networks was not solely the result of the degree distribution heterogeneity of the networks, we applied the core–periphery algorithm as described above to 1,000 surrogate networks, matched for degree distribution, nodes, and edges to the empirical networks. The statistical significance of the core was computed by examining if the size of the largest cliques (constituting the core) in the surrogate networks exceeded the size of the largest cliques in the empirical networks.

### Comparing the cytoarchitecture and efficiency of the core and periphery areas

We used the Brain Connectivity Toolbox (https://sites.google.com/site/bctnet/) [91] for estimating the in- and out-efficiency (based on shortest paths or random walks) of cortical areas. Because of an absence of quantitative information on the strength of connections for the mouse and cat connectomes, these measures were computed in binary connectomes. We used permutation tests for comparing the in- and out-efficiency (based on shortest paths or random walks) and cytoarchitectonic differentiation of the core and periphery areas. The labels of the areas denoting if they belong to the core or the periphery were permuted, and the core–periphery differences were estimated with the Kolmogorov-Smirnov test or the statical energy test, a nonparametric test for comparing two distributions [92] (https://github.com/brian-lau/multdist/blob/master/minentest.m). The procedure was repeated 1,000 times, and the obtained null values were compared to the values obtained with the original core–periphery assignments.

## Supporting information

S1 Fig Presence or absence of cortico-cortical connections in relation to physical distance and cytoarchitectonic similarity. The same relations as in Fig 2 are depicted, but using a different cortico-cortical connectivity dataset for the mouse [21] and the ordinal scale for the macaque monkey cortex as a qualitative measure of the cytoarchitectonic status of cortical areas.
(TIFF)

S2 Fig Multivariate logistic regression relating existence of connections to physical distance and cytoarchitectonic similarity. The depicted values are the regression coefficients obtained from a multivariate model with existence of connections as the dependent variable and physical distance and cytoarchitectonic similarity as two regressors. In all species, physical distance is significantly related to the existence of connections. In all species, apart from the marmoset monkey, cytoarchitectonic similarity relates to existence of connections. Bars correspond to standard errors of the regression coefficients. Note that physical distance and cytoarchitectonic similarity values were linearly rescaled to the 0–1 interval in order to render the corresponding regression coefficients values comparable.
(TIFF)

S3 Fig Robustness of the relation of cytoarchitectonic similarity and existence of connections. (A) The depicted values (mean and standard deviation over 100 reassignments at each level) are the regression coefficients obtained from a multivariate model with existence of connections as a dependent variable and physical distance and cytoarchitectonic similarity as the two regressors. Only cytoarchitectonic similarity coefficients are depicted. Cortical types were reassigned to areas; for instance, if a cortical type was 2, it was randomly reassigned to 1 or 3. The x-axis depicts the percentage of areas that were reassigned to a cortical type. Note that the coefficients remain well above chance even when 80% of the areas were subject to reassignment. (B) Same as in (A), but for reassignments that could stretch the upper limit of the ordinal scale; that is, if an area has cortical type 5, it could be reassigned to level 6. Note that the coefficients remain above null values even when 80% of the areas were subject to reassignment. Null values for the coefficient values were assessed with permutations.
(TIFF)

S4 Fig Cytoarchitectonic similarity relates to the existence of connections in a species-specific manner. The same relations as in Fig 3 are depicted, but using a different cortico-cortical connectivity dataset for the mouse [21] and the ordinal scale for the macaque monkey cortex as a qualitative measure of the cytoarchitectonic status of cortical areas.
(TIFF)

S5 Fig Predictions of quantitative laminar connection origins in the macaque monkey with cortical types. The same relations as in Fig 4 are depicted, but using the ordinal scale (cortical types) for the macaque monkey cortex as a qualitative measure of the cytoarchitectonic status of cortical areas.
(TIFF)

S6 Fig Predictions of quantitative laminar connection origins in macaque monkey with the exclusion of less differentiated parts of the cortex. The same relations as in S5 Fig are depicted, but excluding the cytoarchitectonically less differentiated insular and cingulate cortical areas.
(TIFF)

S7 Fig Predictions of qualitative laminar connection origins in the cat. The same relations as in Fig 5 are depicted, but using (A) binary qualitative classification of connections (“feedforward” and “feedback”) in the cat cortex [88]. (B) The same pattern is observed as in Fig 5B; that is, the cytoarchitecture-based model leads to better predictions than the rostrocaudal-based model, as assessed by the AUC of receiver operating characteristic curves. The conjoint use of the cytoarchitectonic information and the rostrocaudal distances did not lead to statistically significant higher AUC curves compared to the AUC curves of the cytoarchitecture-based model (*p* > 0.05, permutation tests). AUC, area under the curve.
(TIFF)

S8 Fig Robustness of the difference of cytoarchitecture between the core and periphery areas. (A) The depicted values (mean and standard deviation over 100 reassignments at each level) are the statistical energy values for the cytoarchitectonic difference of the core and periphery areas. Cortical types were reassigned to areas; for instance, if a cortical type was 2, it was randomly reassigned to 1 or 3. The x-axis depicts the percentage of areas that were reassigned to a cortical type. (B) Same as in (A), but for reassignments that could stretch the upper limit of the ordinal scale; that is, if an area has cortical type 5, it could be reassigned to level 6. Note that in both cases, the statistical energy values remain above the null values even when 80% of the areas were subject to reassignment. Note that this control analysis was only performed for the cat, since the mouse core and periphery did not exhibit significant cytoarchitectonic differences in the original analysis. Null values for the statistical energy test were assessed with permutations.
(TIFF)

S1 Table Acronyms and full names of cortical areas. Acronyms and full names are listed for the mouse, marmoset monkey, cat, and macaque monkey.
(XLSX)

S2 Table Correspondence of the Desikan-Killiany atlas and the von Economo and Koskinas atlas. Cortical areas of the von Economo and Koskinas atlas [37] are assigned to the Desikan-Killiany atlas [48].
(XLSX)

S3 Table Core areas of the cat connectome. Cortical areas that participate in the largest cliques (C1–C2) of the cat connectome constitute the network core. Whether or not an area participates in each of the largest cliques is denoted by “1” and “0”, respectively.
(XLSX)

S4 Table Core areas of the marmoset monkey connectome. Cortical areas that participate in the largest cliques (C1–C12) of the marmoset monkey connectome constitute the network core. Whether or not an area participates in each of the largest cliques is denoted by “1” and “0”, respectively.
(XLSX)

## Abbreviations

D: dorsal
D/M: dorsal/medial
LR: likelihood ratio
max: maximum
min: minimum
NSG%: percentage of supragranular labeled neurons
V: ventral
V/L: ventral/lateral
