# Supplementary figures and images for: A blueprint of mammalian cortical connectomes
Source: PLoS Biol. 2019 Mar 22;17(3):e2005346. doi: 10.1371/journal.pbio.2005346 (PMC6456226; doi:10.1371/journal.pbio.2005346)

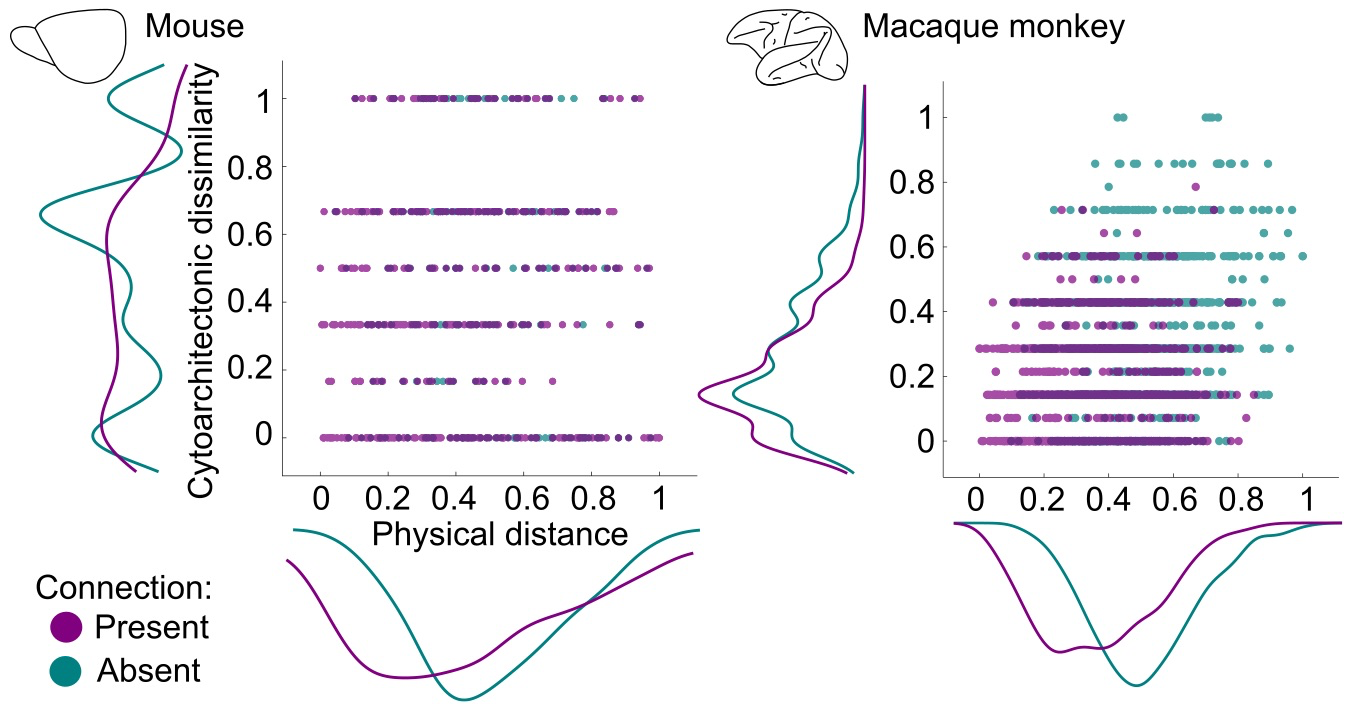

Supplement: S1 Fig — The same relations as in Fig 2 are depicted, but using a different cortico-cortical connectivity dataset for the mouse [21] and the ordinal scale for the macaque monkey cortex as a qualitative measure of the cytoarchitectonic status of cortical areas. (TIFF) [file pbio.2005346.s001.tiff]

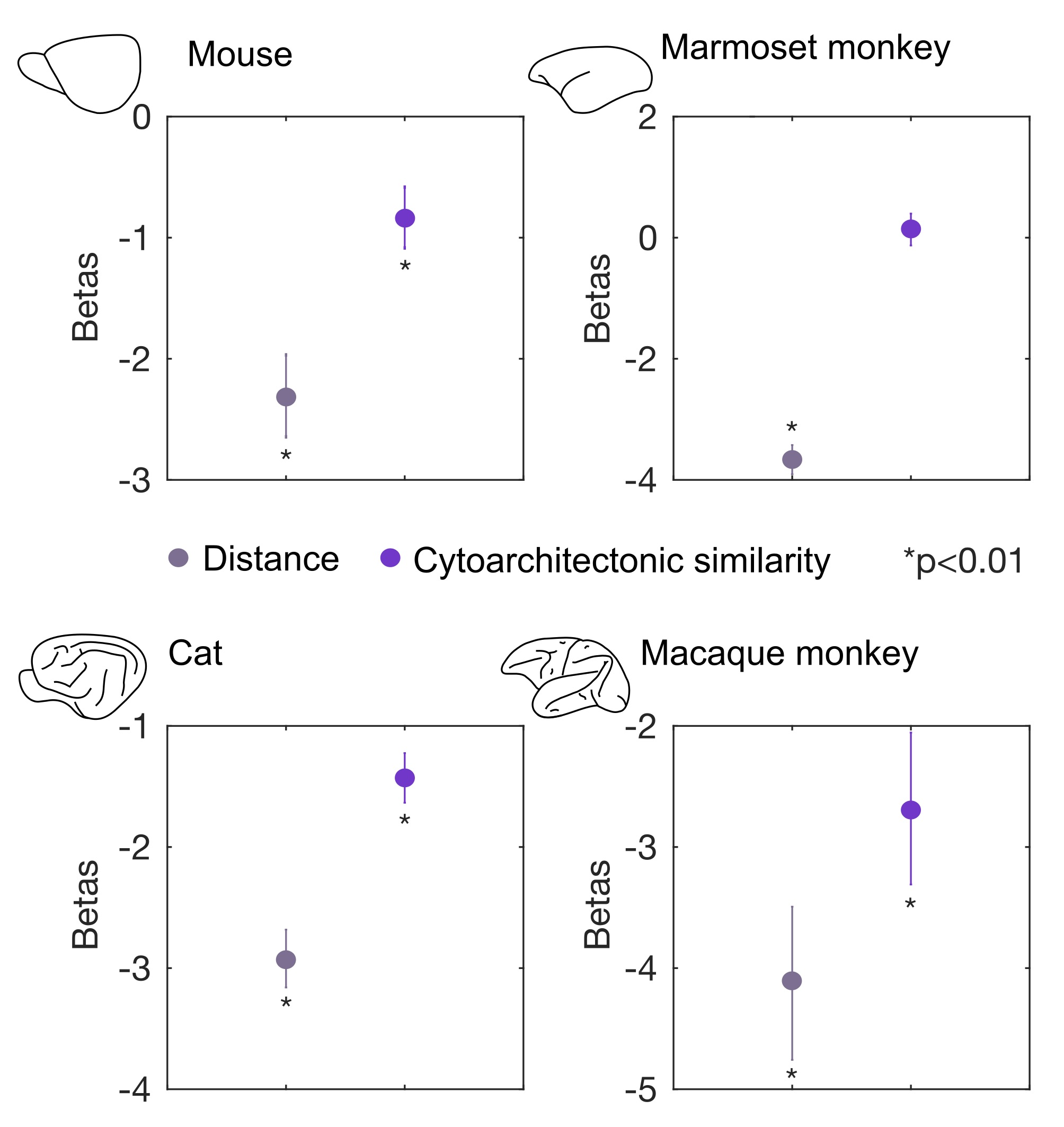

Supplement: S2 Fig — The depicted values are the regression coefficients obtained from a multivariate model with existence of connections as the dependent variable and physical distance and cytoarchitectonic similarity as two regressors. In all species, physical distance is significantly related to the existence of connections. In all species, apart from the marmoset monkey, cytoarchitectonic similarity relates to existence of connections. Bars correspond to standard errors of the regression coefficients. Note that physical distance and cytoarchitectonic similarity values were linearly rescaled to the 0–1 interval in order to render the corresponding regression coefficients values comparable. (TIFF) [file pbio.2005346.s002.tiff]

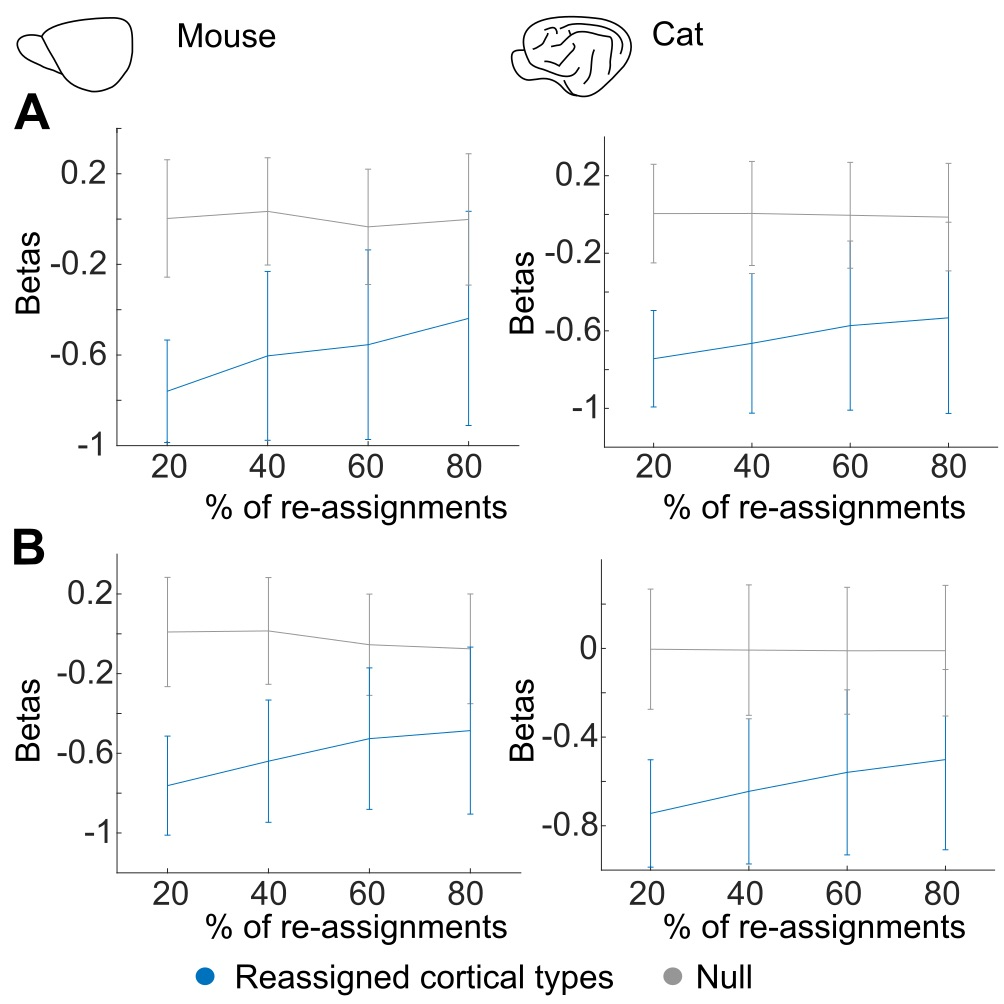

Supplement: S3 Fig — (A) The depicted values (mean and standard deviation over 100 reassignments at each level) are the regression coefficients obtained from a multivariate model with existence of connections as a dependent variable and physical distance and cytoarchitectonic similarity as the two regressors. Only cytoarchitectonic similarity coefficients are depicted. Cortical types were reassigned to areas; for instance, if a cortical type was 2, it was randomly reassigned to 1 or 3. The x-axis depicts the percentage of areas that were reassigned to a cortical type. Note that the coefficients remain well above chance even when 80% of the areas were subject to reassignment. (B) Same as in (A), but for reassignments that could stretch the upper limit of the ordinal scale; that is, if an area has cortical type 5, it could be reassigned to level 6. Note that the coefficients remain above null values even when 80% of the areas were subject to reassignment. Null values for the coefficient values were assessed with permutations. (TIFF) [file pbio.2005346.s003.tiff]

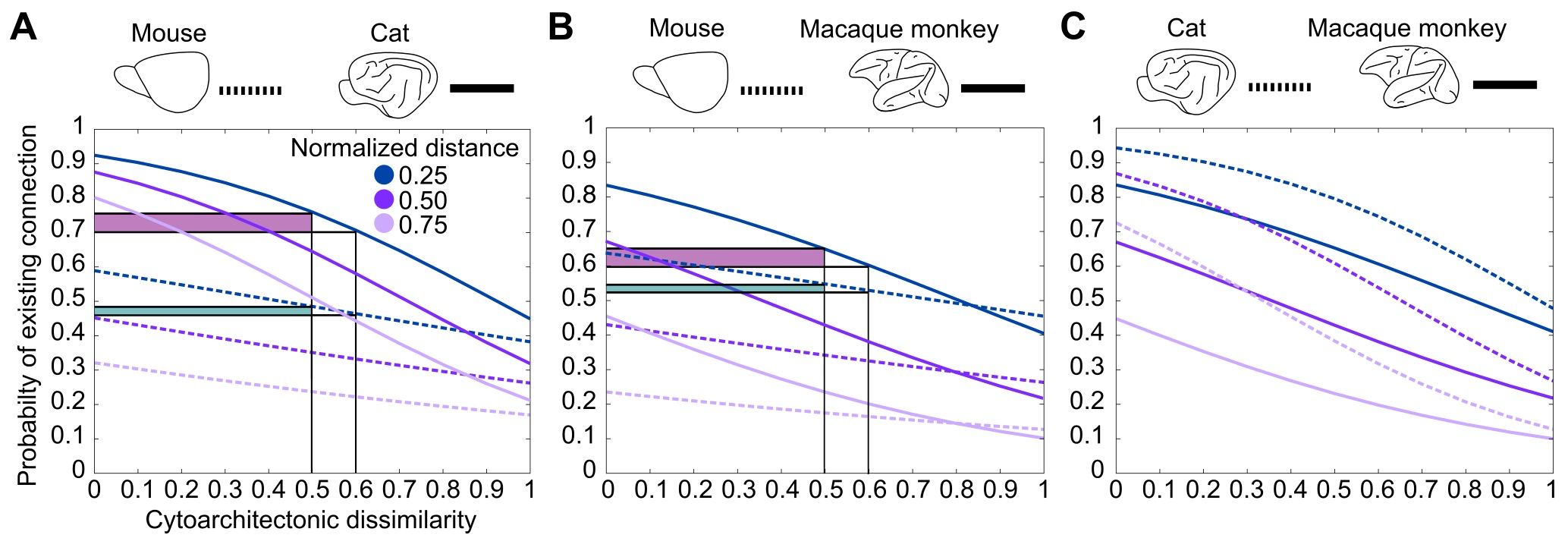

Supplement: S4 Fig — The same relations as in Fig 3 are depicted, but using a different cortico-cortical connectivity dataset for the mouse [21] and the ordinal scale for the macaque monkey cortex as a qualitative measure of the cytoarchitectonic status of cortical areas. (TIFF) [file pbio.2005346.s004.tiff]

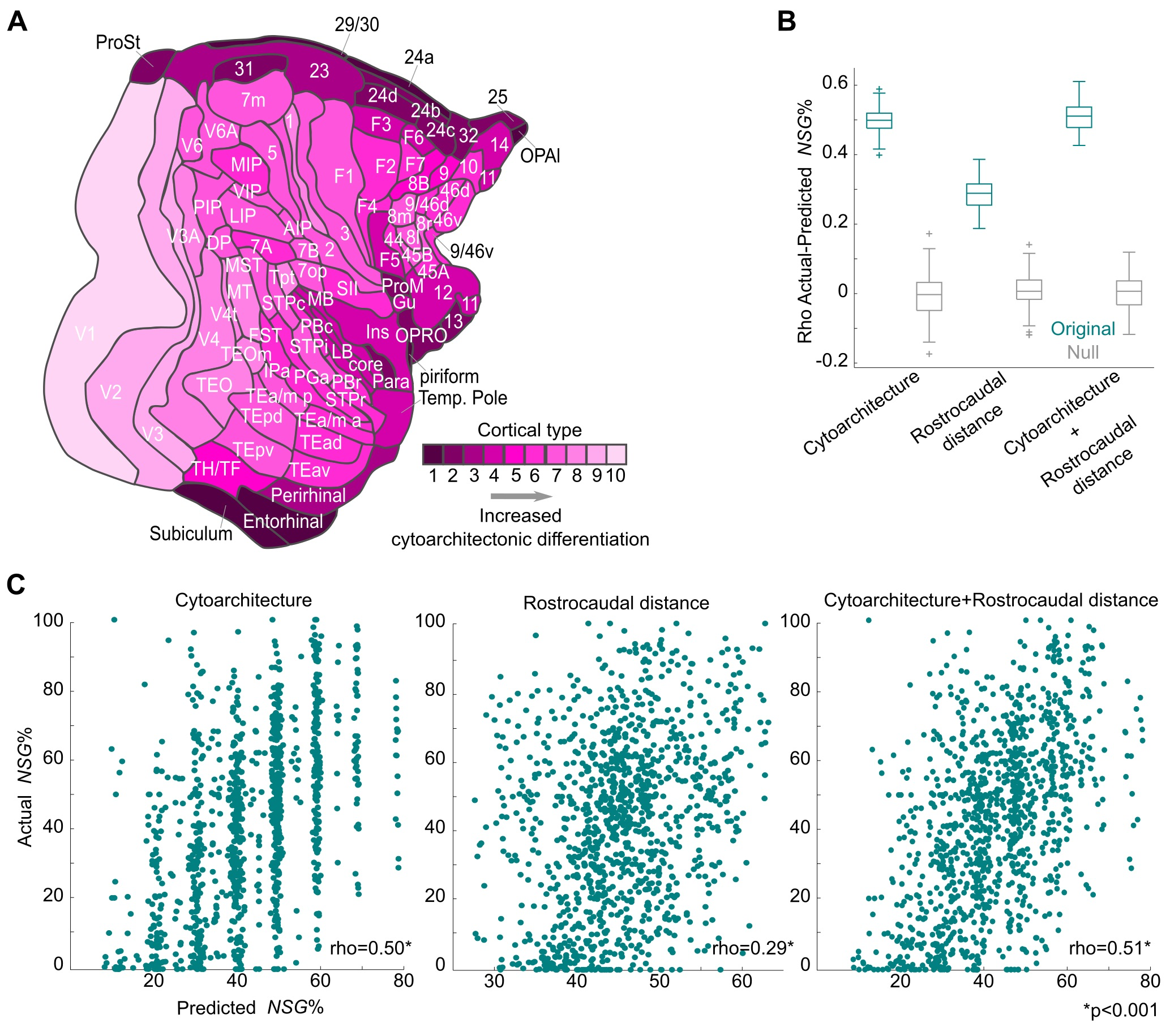

Supplement: S5 Fig — The same relations as in Fig 4 are depicted, but using the ordinal scale (cortical types) for the macaque monkey cortex as a qualitative measure of the cytoarchitectonic status of cortical areas. (TIFF) [file pbio.2005346.s005.tiff]

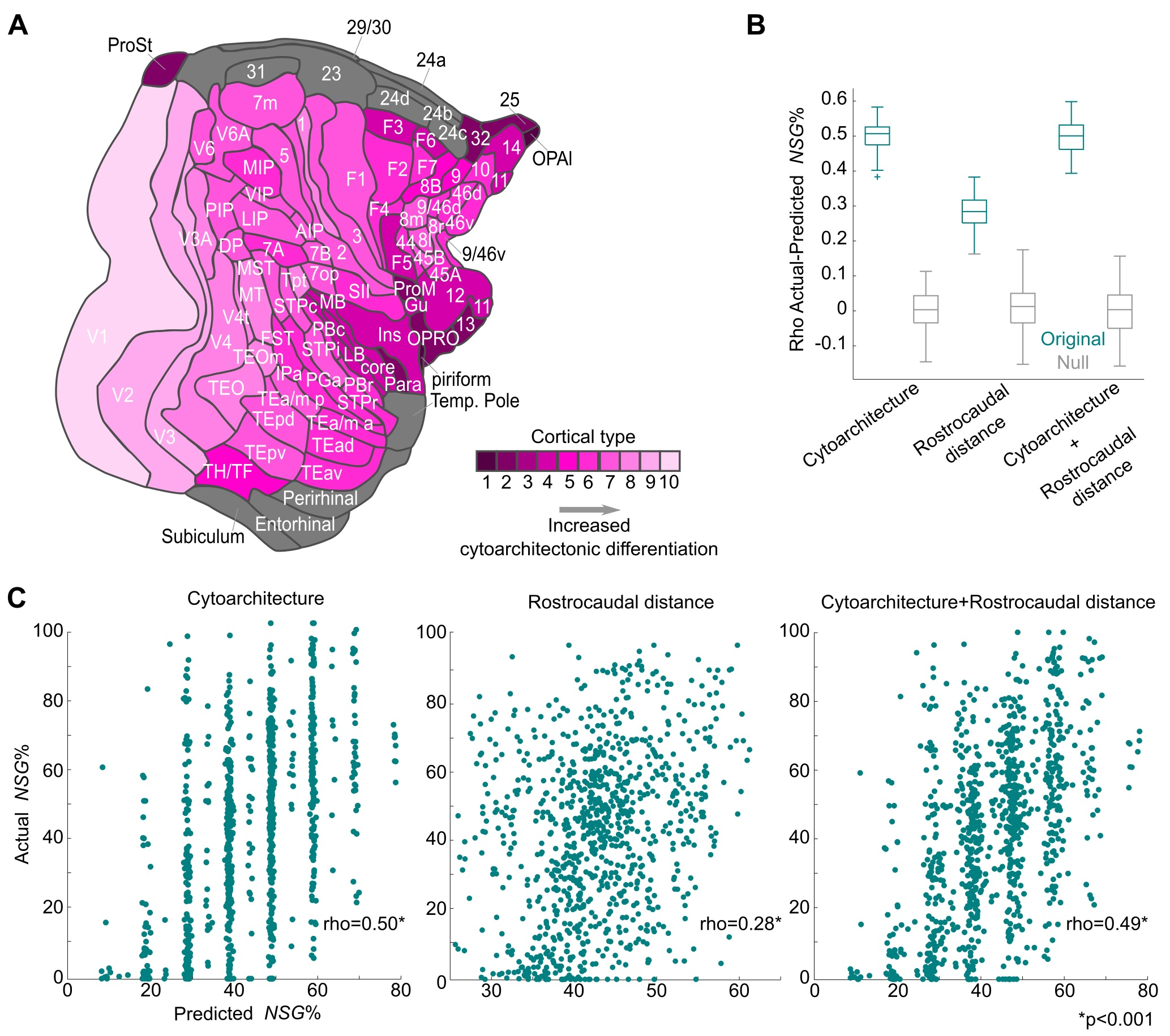

Supplement: S6 Fig — The same relations as in S5 Fig are depicted, but excluding the cytoarchitectonically less differentiated insular and cingulate cortical areas. (TIFF) [file pbio.2005346.s006.tiff]

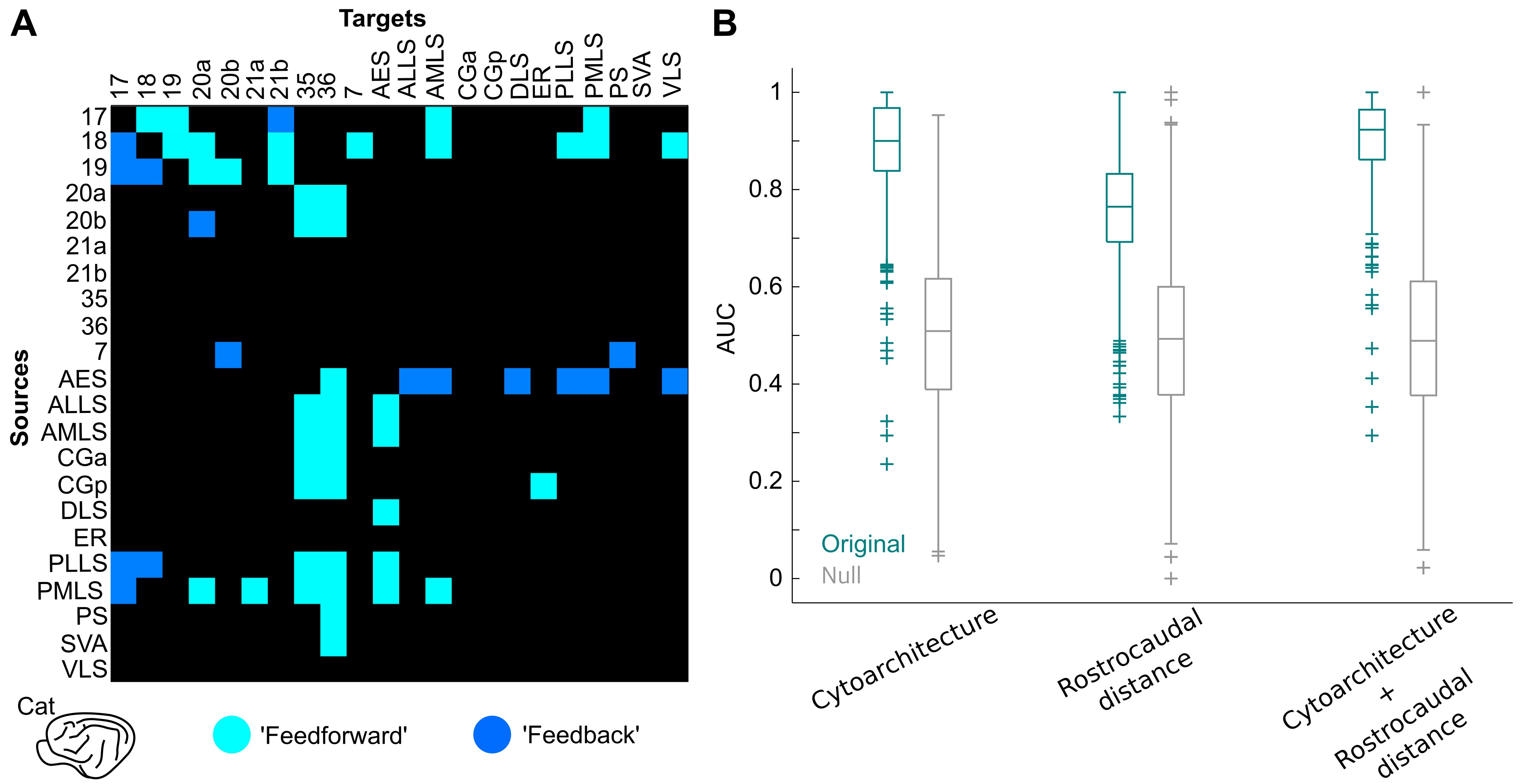

Supplement: S7 Fig — The same relations as in Fig 5 are depicted, but using (A) binary qualitative classification of connections (“feedforward” and “feedback”) in the cat cortex [88]. (B) The same pattern is observed as in Fig 5B; that is, the cytoarchitecture-based model leads to better predictions than the rostrocaudal-based model, as assessed by the AUC of receiver operating characteristic curves. The conjoint use of the cytoarchitectonic information and the rostrocaudal distances did not lead to statistically significant higher AUC curves compared to the AUC curves of the cytoarchitecture-based model (p > 0.05, permutation tests). AUC, area under the curve. (TIFF) [file pbio.2005346.s007.tiff]

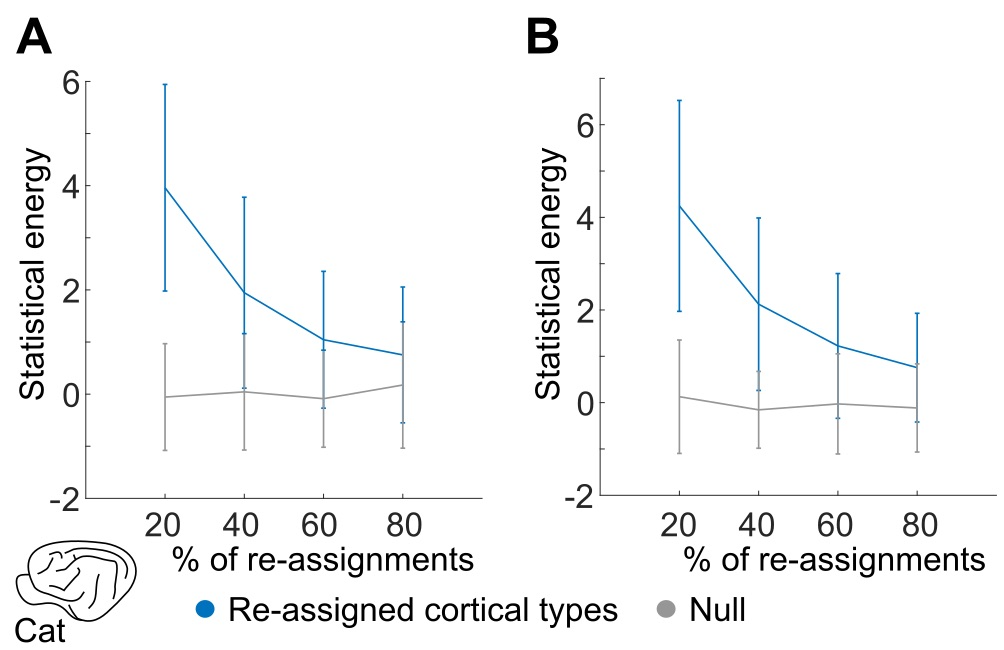

Supplement: S8 Fig — (A) The depicted values (mean and standard deviation over 100 reassignments at each level) are the statistical energy values for the cytoarchitectonic difference of the core and periphery areas. Cortical types were reassigned to areas; for instance, if a cortical type was 2, it was randomly reassigned to 1 or 3. The x-axis depicts the percentage of areas that were reassigned to a cortical type. (B) Same as in (A), but for reassignments that could stretch the upper limit of the ordinal scale; that is, if an area has cortical type 5, it could be reassigned to level 6. Note that in both cases, the statistical energy values remain above the null values even when 80% of the areas were subject to reassignment. Note that this control analysis was only performed for the cat, since the mouse core and periphery did not exhibit significant cytoarchitectonic differences in the original analysis. Null values for the statistical energy test were assessed with permutations. (TIFF) [file pbio.2005346.s008.tiff]
